# Identification of driver modules in pan-cancer via coordinating coverage and exclusivity

**DOI:** 10.18632/oncotarget.16433

**Published:** 2017-03-21

**Authors:** Bo Gao, Guojun Li, Juntao Liu, Yang Li, Xiuzhen Huang

**Affiliations:** ^1^ School of Mathematics, Shandong University, Jinan, Shandong, 250100, China; ^2^ Department of Computer Science, Arkansas State University, Jonesboro, Arkansas, 72401, USA; ^3^ Molecular Biosciences Program, Arkansas State University, Jonesboro, Arkansas, 72401, USA

**Keywords:** pan-cancer, coverage, exclusivity, driver gene, network module

## Abstract

It is widely accepted that cancer is driven by accumulated somatic mutations during the lifetime of an individual. Cancer mutations may target relatively small number of cell functional modules. The heterogeneity in different cancer patients makes it difficult to identify driver mutations or functional modules related to cancer. It is biologically desired to be capable of identifying cancer pathway modules through coordination between coverage and exclusivity. There have been a few approaches developed for this purpose, but they all have limitations in practice due to their computational complexity and prediction accuracy. We present a network based approach, CovEx, to predict the specific patient oriented modules by 1) discovering candidate modules for each considered gene, 2) extracting significant candidates by harmonizing coverage and exclusivity and, 3) further selecting the patient oriented modules based on a set cover model. Applying CovEx to pan-cancer datasets spanning 12 cancer types collecting from public database TCGA, it demonstrates significant superiority over the current leading competitors in performance. It is published under GNU GENERAL PUBLIC LICENSE and the source code is available at:https://sourceforge.net/projects/cancer-pathway/files/

## INTRODUCTION

With the rapid progress of next generation sequencing technologies, a huge amount of mutation data of thousands of patients for dozens of cancer types has become available [1–5]. The mutational heterogeneity in different cancer patients brings challenges in distinguishing driver mutations, which contribute to tumorigenesis, from sporadic, passenger mutations [6] and, in identifying driver pathway modules whose behavior perturbation would lead to tumorigenesis [7, 8].

There have been a few approaches developed to discover driver genes and pathways. For example, some of them were designed to identify mutated genes or regions of copy number alterations based on empirically derived background alteration rates [9]. With the prior biological knowledge, such as KEGG pathways [10] or GO functional groups [11], significantly mutated known pathways and functional modules were identified by some statistical methods [12, 13]. However, due to the incomplete knowledge of protein interactions and pathways in human, these methods have not been able to accurately detect novel pathway modules that are targeted by cancer mutations.

*De novo* analysis of driver pathway modules in cancer is important to obtain novel biological discoveries. According to the existing knowledge, a driver pathway module usually exhibits two combinatorial properties: high coverage and high exclusivity [1, 2, 14, 15]. High coverage means that most patients have at least one mutated gene in the module. High exclusivity means that most patients have only one mutated gene in that module. Some approaches were developed to identify gene modules with the two properties. RME [16] calculated the exclusivity weight as the percentage of covered patients that contain exactly one mutation within a gene set. Another exclusive metric, Dendrix weight [17], was defined as the difference of coverage and coverage overlap of a gene set (see Methods Section for details). Based on Dendrix weight, Vandin et al. proposed a greedy algorithm and a Markov chain Monte Carlo (MCMC) algorithm [17], and Zhao et al. introduced MDPFinder including a binary linear programming (BLP) method and a genetic algorithm [18] to identify large weight modules. Compared to MCMC algorithm, the BLP method is more efficient. A multi-objective optimization model based on a Genetic Algorithm (MOGA) was introduced to adjust the trade-off between coverage and exclusivity [19]. Multi-Dendrix [20] designed a new metric as the sum of Dendrix weights and adopted a new programming model to identify multiple modules simultaneously. CoMDP proposed an exact mathematical programming method to identify co-occurring mutated driver pathways [21]. However, the mutational landscape of cancer usually consists of genes of which some are mutated frequently and rarely for others, making the exclusive metrics employed in these methods unable to handle this broad spectrum of mutational frequencies properly. It has been observed that genes with high mutation frequencies dominate the Dendrix weight and the majority of the coverage comes from one gene in the identified modules [22]. Biologically, genes in a functional module should be closely correlated in pathway or protein-protein interaction (PPI) networks. Gene sets identified without consideration of pathways or PPI networks may not be correlated and thus not necessarily to form a driver module.

Resorting to the increasing knowledge of pathways and PPI networks, the approaches for identification of driver genes or pathway modules over PPI networks should be developed. To integrate two datasets together, one fast and reliable method was presented in [23]. A combinatorial model was proposed for global module detection in complex networks [24]. HotNet2 [25] is a network based method that delves into the long tail of rarely mutated genes and finds mutated subnetworks. MEMo [26] and MEMCover [27] are both network based methods to systematically identify mutually exclusive network modules. MEMo outputs the significantly exclusive modules evaluated by a random permutation testing method. MEMCover evaluates the mutual exclusivity degree for gene pairs with random permutation testing method. Both MEMo and MEMCover only consider those gene pairs representing interactions in a PPI network, which restricts the discovered networks to existing interaction networks. Furthermore, the random permutation testing method is computationally expensive for scoring the mutual exclusivity degree of gene sets.

Considering the limitation of current combinatorial evaluation metric and random permutation testing method, a couple of novel probabilistic models evaluating mutual exclusivity for gene modules have been developed, such as muex [28], mutex [29], CoMEt [22], WeSME [30], WExT [31], etc. Those methods overcome some of the drawbacks of previous exclusivity evaluation method. However, compared to the combinatorial evaluation metric, the probabilistic methods are too complicated to efficiently calculate the exclusive scores for gene modules. For example, to reduce the computational complexity, the mutex algorithm limited the search space to genes having a common downstream signaling target only [29]. A combinatorial evaluation metric overcoming the limitation of current combinatorial metrics would be desirable and may be applied to search for mutually exclusive modules to a much larger scale.

A few methods for predicting driver genes/pathways through integrating expression data, sequence information, structural information, functional annotation and biomedical literature, aiming at improving their prediction accuracy, have also been developed [32–36]. Furthermore, the commonalities and specificities of driver gene sets among multiple cancer types have been systematically investigated in [37]. More comprehensive reviews can be referred to [38–41].

**Figure 1:**
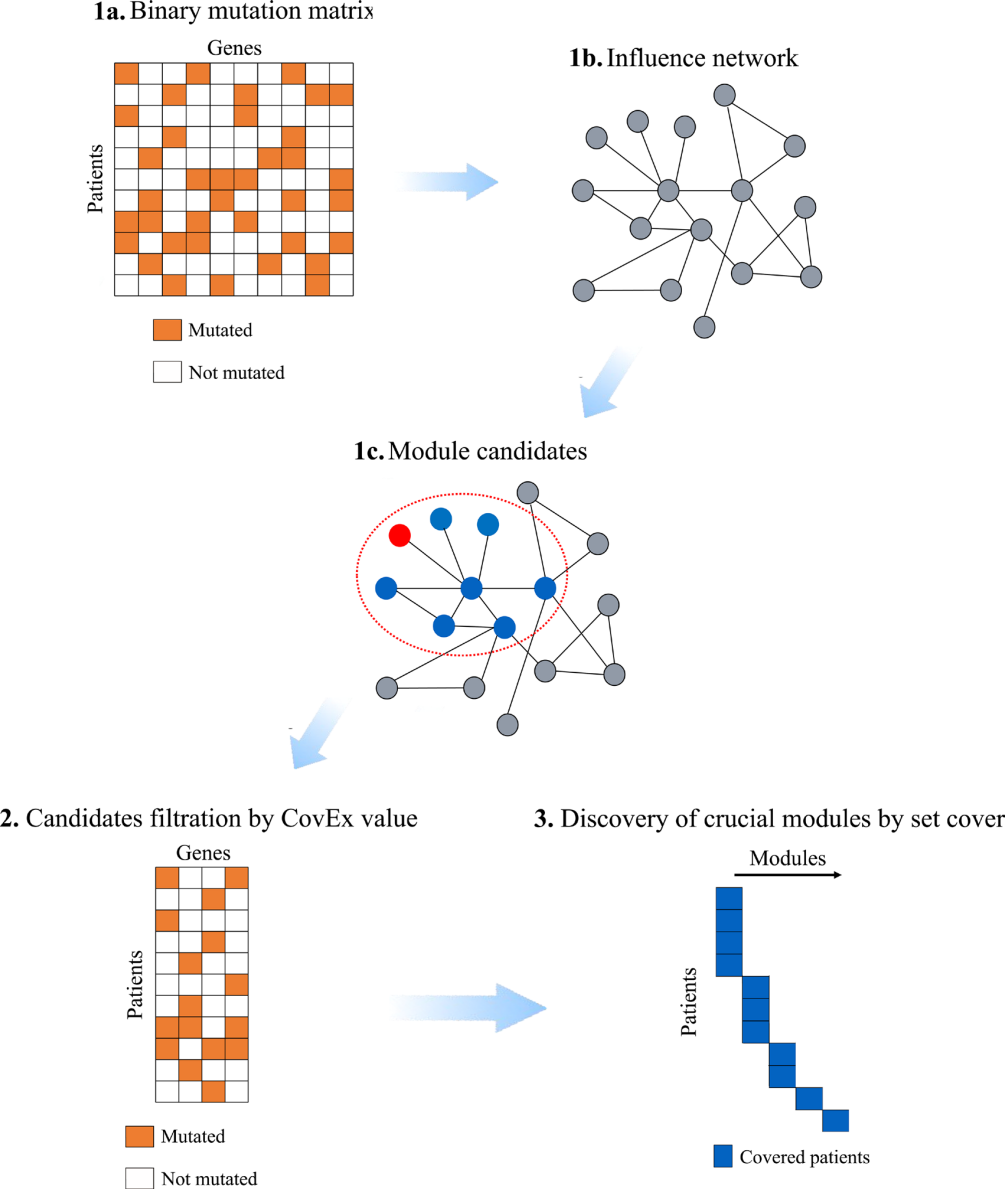
Flowchart of CovEx In the first phase, a binary mutation matrix is built according to the mutation data; an influence network containing the mutated genes only is constructed based on an annotated PPI network; each candidate module is identified within a local influence network rooted at a node. In the second phase, the CovEx value for each candidate module is calculated and modules with small CovEx values are filtered. In the third phase, a minimum set cover model is applied to identify those patient specific crucial modules.

Our extensive studies have revealed that all the existing tools developed for identification of cancer genes/pathways perform poorly with low accuracies and highly inconsistent solutions. In this paper, we present a network-based algorithm to identify exclusive network modules. The algorithm consists of three phases. In the first phase, we exhaustively enumerate gene sets for each considered gene in a local constructed influence network. To be specific, we search for the candidate modules by optimizing Dendrix weight on local networks each roots at a node across an influence network which measures the topological relationships between genes in the dataset. As we previously mentioned, the candidate modules identified in the first phase may not be exclusive at all. In the second phase, by a new designed combinatorial coverage and exclusivity evaluation metric, CovEx, which overcomes the limitation of Dendrix weight (see Methods Section for details), we filter out those candidate modules with poor coverage and exclusivity property. After the first two phases, only significant candidate modules were left. Biologically, each patient should have at least one driver module, and the driver module for different patients may be different. To identify the specific driver module for each patient, we employ a minimum set cover model to select the specific patient oriented driver module in the third phase. Obviously, the modules selected in the third phase may be critical and are most likely to be the desired pathway modules. The flowchart of our method, CovEx, is plotted in Figure 1. We applied CovEx to three annotated PPI networks which are the same as used in [25]. We ran CovEx on each of the three PPI networks to get eight solutions by resetting parameter eight times, and therefore getting 24 predictions in total on the three networks. To further refine the CovEx's predictions and elucidate the crosstalk of identified modules, we applied a new designed consensus method to the 24 CovEx predictions.

We tested the CovEx by comparing it with other competitors based on the refined predictions. The comparison results demonstrate that it outperforms other competitors in terms of annotated cancer genes. In addition, CovEx is flexible for users to control the number of genes to be output against others. For example, CovEx outputs consensus modules containing 88 genes with 66 being annotated NCG cancer genes, denoted by “88 with 66” for short, 140 with 90, and 236 with 124, respectively, v.s. 138 with 60 by HotNet2, a state-of-the-art tool, and 82 with 46 by MSEA [33], a tool developed by incorporating different biological knowledge, e.g. annotations of protein domain structures. Therefore, the accuracy has been improved from 43% of HotNet2 to 75% of CovEx, and even for MSEA, only 56% can be reached, while the sensitivity has also been improved to some extent. One more ingredient for CovEx lies in the new effective combinatorial metric which subtly harmonizes coverage and exclusivity. The functional analysis of the refined network modules has also brought some new insights into the cancer related pathways and other functional modules.

## RESULTS

We downloaded the aberration datasets as well as the influence matrix files for the three PPI networks: HINT+HI2012, iRefIndex and Multinet from HotNet2 website (http://compbio-research.cs.brown.edu/pancancer/hotnet2/). The pan-cancer datasets span 12 cancer types [25] which consist of bladder urothelial carcinoma (BLCA), breast invasive carcinoma (BRCA), colon adenocarcinoma and rectum adenocarcinoma (COADREAD, COAD and READ are combined into one type), glioblastoma multiforme (GBM), head and neck squamous cell carcinoma (HNSC), kidney renal clear cell carcinoma (KIRC), acute myeloid leukemia (LAML), lung adenocarcinoma (LUAD), lung squamous cell carcinoma (LUSC), ovarian serous cystadenocarcinoma (OV), uterine corpus endometrioid carcinoma (UCEC). The somatic single nucleotide variants (SNVs), small indels and copy number aberrations (CNAs) data were combined together to be considered simultaneously. One pan-cancer dataset contains 3,110 patients including 19,457 genes. The dataset after filtration with RNA-seq expression data includes 11,565 genes. The datasets after and before filtrating are denoted by pan-cancer datasets A and B, respectively. Another pan-cancer dataset with potential germline mutations removed including 11533 genes is denoted as pan-cancer dataset C. To evaluate our results, we collected annotated cancer genes as comparison benchmarks from different publicly available sources where 1,571 protein-coding cancer genes were downloaded from NCG 5.0 [42], 571 genes were downloaded from Cancer Gene Census [43], and finally 138 cancer genes were downloaded from [44]. The three benchmarks of cancer genes are referred to as NCG, CGC and 20/20 rule cancer genes, respectively.

For each pan-cancer dataset, we applied CovEx with parameters (λ, *k*), λ = 0, 1; *k* = 2, 3, 4, 5 to each of the three PPI networks, HINT+HI2012, iRefIndex and Multinet, and obtained 24 predictions each consists of exclusive network modules. The parameter λ controls the exclusivity degree in Dendrix weight and *k* corresponds to the identified module size (see Methods Section for details). If no otherwise specified, the pan-cancer dataset A and the NCG cancer genes are selected as the dataset and the cancer gene benchmark in the following. For convenience, we refer to the number of identified cancer genes as sensitivity and the ratio of number of identified cancer genes to that of the predicted as accuracy.

### Results from single solutions

**Figure 2:**
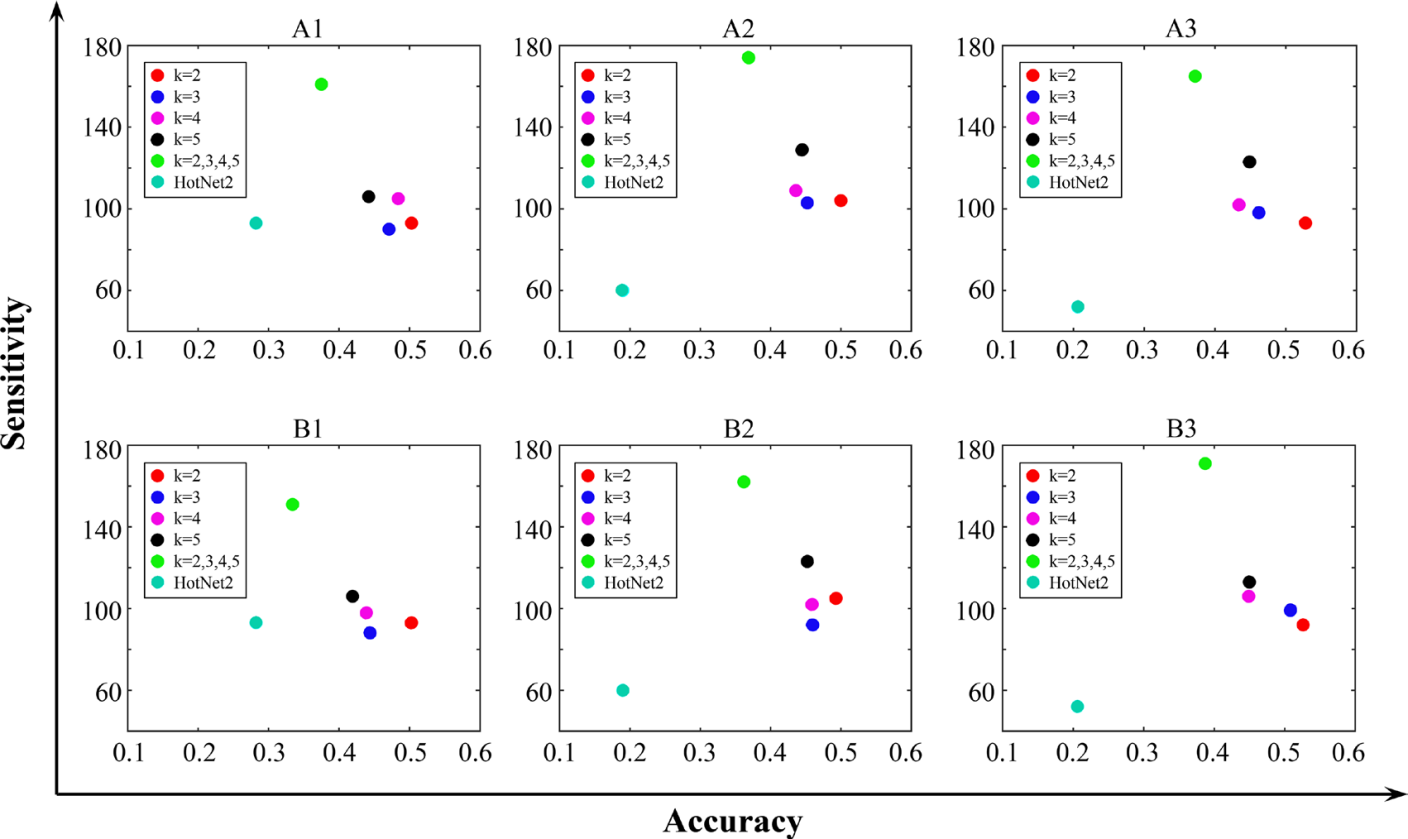
Comparison Results of CovEx and HotNet2 The HotNet2 results are obtained based on the mutation frequency score on the same dataset and PPI networks. **A1**, **B1** are obtained on HINT+HI2012; **A2**, **B2** on iRefIndex; and **A3**, **B3** on Multinet. A1, A2, A3 are obtained with λ=0; and B1, B2, B3 with λ *=* 1. *k* = 2, *k* = 3, *k* = 4, *k* = 5 correspond to different single CovEx predictions, and *k* = 2, 3, 4, 5 corresponds to CovEx predictions which are obtained by combining the four predictions for specific λ and PPI network.

The number of modules identified by the CovEx ranges from 70 to 129 of the 24 predictions (Supplementary Section 1, Supplementary Tables 1, 2). Especially, we analyzed the sensitivity and accuracy of each prediction. Considering the 8 predictions for each PPI network, we found their minimum sensitivities of 88, 92 and 92 with the corresponding accuracies of 44.4%, 46.0% and 52.6%, and their maximum sensitivities of 106, 129 and 123 with the corresponding accuracies of 44.2%, 44.5% and 44.9% on HINT+HI2012, iRefIndex and Multinet, respectively. We further observed that the minimum and maximum accuracies in all the 24 predictions are 41.9% and 52.8%, respectively, against 8.0% in the original dataset. Combining all the 24 predictions, we obtained 1110 genes with 288 cancer genes, and thus the accuracy of 25.9% smaller than that of each single prediction because the false positives identified in the 24 respective predictions are highly inconsistent. Furthermore, we found much higher accuracies for the case where *s*-modules are filtered for *s* = 1, 2, 3, respectively (Supplementary Section 2, Supplementary Tables 3, 4, 5, 6, Supplementary Figure 1). The comparison results from Figure 2 demonstrate that CovEx substantially outperforms HotNet2 no matter which parameter pair is used (Supplementary Sections 3, 4).

### Results from consensus solution

As we see in the last section, the accuracies of the 24 single predictions are similar, but the accuracy of the combined prediction is much smaller because the false positives across the different predictions are highly inconsistent. To get a more accurate prediction, we applied the consensus method described in Methods Section to the 24 individual predictions. We obtained 15 sub-network modules of type 1 and 32 of type 2, containing 236 genes totally with 124 cancer genes (Supplementary Tables 7, 8). We compared all the sub-network modules with KEGG pathway and GO database in STRING v10 [45].

### The type 1 sub-networks

The largest type 1 sub-network (network index 1, Supplementary Table 7) contains 121 genes with 78 cancer genes. After functional enrichment analysis, we found that 88 KEGG pathways, 946 biological process GO-terms, 90 molecular function GO-terms and 75 cellular component GO-terms are significantly enriched. The most significantly enriched KEGG pathway is the cancer pathway (pathway ID 05200) with the false discovery rate of 1.32e-26. The functional enrichment analysis identifies the critical pathways and GO terms related to cancer. The large number of significantly enriched functional modules exhibits the crosstalk between different functional modules. The second largest type 1 sub-network (network index 5, Supplementary Table 7) contains 11 genes with 6 cancer genes. We found that 38 KEGG pathways, 156 biological process GO-terms, 1 molecular function GO-terms and 7 cellular component GO-terms are significantly enriched. The most significantly enriched KEGG pathway is MicroRNAs in cancer (pathway ID 05206). Comparing CovEx predictions to HotNet2 predictions, we found that some of the annotated cancer genes, e.g. BAP1, EGFR, ERBB4, KRAS, NRAS, WT1, etc. were identified by both CovEx and HotNet2 in the largest type 1 sub-network, while some of them, e.g. ABL1, AKT1, APC, RB1, etc. were identified only by CovEx. These cancer genes have been known playing important roles in many types of process. For example, mutations in EGFR are associated with lung cancer. ABL1 plays a role in many key processes linked to cell growth and survival such as cytoskeleton remodeling in response to extracellular stimuli, cell motility and adhesion, receptor endocytosis, autophagy, DNA damage response and apoptosis. AKT1 is one of 3 closely related serine/threonine-protein kinases (AKT1, AKT2 and AKT3) called the AKT kinase which regulates many processes including metabolism, proliferation, cell survival, growth and angiogenesis. APC is a tumor suppressor which promotes rapid degradation of CTNNB1 and participates in Wnt signaling as a negative regulator. APC activity is correlated with its phosphorylation state, activates the GEF activity of SPATA13 and ARHGEF4, plays a role in hepatocyte growth factor (HGF)-induced cell migration, etc. We also identified genes which do not belong to any of the cancer gene benchmarks, such as CRIPAK, INTS4, PARP10, etc. It has been suggested that the loss of CRIPAK in breast tumors might contribute to hormonal independence. INTS4 is a component of the Integrator complex, a complex involved in the small nuclear RNAs (snRNA) U1 and U2 transcription and in their 3′-box-dependent processing. PARP10 may play a role in cell proliferation and be required for the maintenance of cell cycle progression. More information can be referred to Supplementary Section 5.

Among the 11 genes of the second largest type 1 sub-network, NOTCH1, NOTCH3, NOTCH4, SPOP and PTEN were also identified by HotNet2. Both NOTCH1 and NOTCH3 function as a receptor for membrane-bound ligands Jagged1, Jagged2 and Delta1 to regulate cell-fate determination and affect the implementation of differentiation, proliferation and apoptotic programs. PTEN is a well-known tumor suppressor gene. PTEN acts as a dual-specificity protein phosphatase, dephosphorylating tyrosine-, serine- and threonine-phosphorylated proteins. SPOP is a component of a cullin-RING-based BCR (BTB-CUL3-RBX1) E3 ubiquitin-protein ligase complex that mediates the ubiquitination of target proteins, leading most often to their proteasomal degradation. CovEx identified extra NCG cancer genes MECOM and NF1 and CGC cancer gene IKBKB. MECOM is the complex locus of MDS1 and EVI1, and NF1 stimulates the GTPase activity of Ras. NF1 shows greater affinity for Ras GAP, but lower specific activity. Also, NF1 may be a regulator of Ras activity. IKBKB plays an essential role in the NF- kappa-B signaling pathway which is activated by multiple stimuli such as inflammatory cytokines, bacterial or viral products, DNA damages or other cellular stresses. IKBKB also acts as part of the canonical IKK complex in the conventional pathway of NF-kappa-B activation and phosphorylates inhibitors of NF-kappa-B on 2 critical serine residues.

There are two sub-networks both containing 8 genes. One sub-network (network index 8, Supplementary Table 7) includes two cancer genes PABPC1, GIGYF2. We found that 3 KEGG pathways, 2 biological process GO-terms, 2 molecular function GO-terms and 1 cellular component GO-term are significantly enriched. Especially, the significantly enriched cellular component GO-term is cytosol (GO:0005829) containing 7 identified genes. Among the 8 genes, PABPC1 is a NCG cancer gene which may be involved in cytoplasmic regulatory processes of mRNA metabolism such as pre-mRNA splicing. Its function in translational initiation regulation can either be enhanced by PAIP1 or repressed by PAIP2. Another NCG cancer gene GIGYF2 may act cooperatively with GRB10 to regulate tyrosine kinase receptor signaling, including IGF1 and insulin receptors. SMG1 which was identified by both CovEx and HotNet2 plays a central role in nonsense-mediated decay (NMD) of mRNAs containing premature stop codons by phosphorylating UPF1/RENT1. MAP1A encodes a structural protein involved in the filamentous cross- bridging between microtubules and other skeletal elements. Phosphorylated MAP1B may play a role in the cytoskeletal changes that accompany neurite extension. The other sub-network (network index 13, Supplementary Table 7) includes two NCG genes CDH1 and COL5A1. Totally, 14 KEGG pathways, 21 biological process GO-terms, 6 molecular function GO-terms and 6 cellular component GO-terms are significantly enriched. CDH1 is involved in mechanisms regulating cell-cell adhesions, mobility and proliferation of epithelial cells, and potentially plays an invasive suppressor role. COL5A1 is a NCG cancer gene acting as a minor connective tissue component of nearly ubiquitous distribution. ITGA2 encodes the alpha subunit of a transmembrane receptor for collagens and related proteins. ITGB2 encodes an integrin beta chain, which combines with multiple different alpha chains to form different integrin heterodimers. The top significantly enriched KEGG pathways and different GO-terms of the above sub-networks can be found in Supplementary Table 9. The network images of the four largest sub-networks of type 1 constructed by STRING v10 can be seen in Supplementary Figures 2–5.

### The type 2 sub-networks

We discovered type 2 sub-networks which are actually induced by all the nodes of weight 2 in the network *N*. The two genes NLRP1 and NLRP3 which are connected by an edge in a type 2 sub-network output by CovEx are NCG cancer genes and belong to NLR family. They are enriched in 1 KEGG pathway, 3 biological process and 1 cellular component GO-terms, such as NOD-like receptor signaling pathway (pathway ID 04621), positive regulation of interleukin-1 beta secretion (GO:0050718), inflammasome complex (GO:0061702). The two genes CNOT1 and CNOT3 were also identified to be involved in a type 2 sub-network, and be enriched in RNA degradation (pathway ID 03018), where CNOT3 is a NCG cancer gene. The pair of genes ITPR1 and ITPR2 were also identified in a type 2 sub-network, and enriched in 24 KEGG pathways, 13 biological processes, 2 molecular functions and 4 cellular component GO-terms. The most significantly enriched pathways or GO terms are Phosphatidylinositol signaling system (pathway ID 04070), inositol phosphate-mediated signaling (GO:0048016), inositol 1,4,5-trisphosphate-sensitive calcium-release channel activity (GO:0005220) and platelet dense tubular network (GO:0031094). It was found that some sub-network modules of type 2 intersected with type 1 sub-networks, such as BACH1 and BACH2 sub-networks. BACH1 and BACH2 are both transcriptional regulators that act as repressor or activator.

### Consensus results comparison of CovEx to HotNet2

The comparison results based on single predictions have demonstrated that CovEx is significantly superior to HotNet2. It has been shown that our consensus method itself is also superior to that used in HotNet2 (Supplementary Section 6, Supplementary Tables 10, 11, 12, Supplementary Figure 6).

We compared the consensus results of CovEx with HotNet2 in terms of sensitivity and accuracy which are commonly used as standard criterion for comparison of biological tools. When pan-cancer dataset A is considered, CovEx predicted 236 genes with 124 cancer genes of accuracy 52.5% by applying the consensus method to all the 24 individual CovEx predictions. In contrast, HotNet2, which is a popular software of same kind, identified 138 genes with 60 cancer genes of the accuracy of 43.5% on pan-cancer dataset A by using the consensus method used in HotNet2 [25]. Compared to results of HotNet2, CovEx identified much more cancer genes with even larger accuracy. For pan-cancer dataset B and C, we also applied CovEx with (λ, *k*), λ = 0, 1; *k* = 2, 3, 4, 5 to each of the three PPI networks, HINT+HI2012, iRefIndex and Multinet, and obtained 24 predictions each consists of exclusive network modules, respectively. After applying the consensus method to the 24 predictions for each pan-cancer dataset, 234 genes with 113 cancer genes and 261 genes with 122 cancer genes are predicted for pan-cancer dataset B and C, with the accuracy of 48.3% and 46.7%, respectively. In contrast, HotNet2 predicted 147 genes with 54 cancer genes and 99 genes with 45 cancer genes for pan-cancer dataset B and C, with the accuracy of 36.7% and 45.5%, respectively. In addition, we applied the consensus method to the 24 individual CovEx predictions with *s*-modules filtered for different value of *s* (see Methods Section for details). It was observed that the accuracy was improved as the parameter *s* increases but at cost of decreasing sensitivity to an extent. For example, 88 genes with 66 cancer genes are predicted by CovEx with the accuracy of 75% when *s* was set to 7. CovEx identified more cancer genes with much larger accuracy than HotNet2. We had the same observations when CGC or 20/20 rule cancer genes were selected as comparison benchmarks (Supplementary Section 7, Supplementary Figure 7).

### Results for single cancer types

We analyzed each dataset of a single cancer type for the pan-cancer dataset A. The numbers of output genes and sensitivities and accuracies of CovEx and HotNet2 for each single cancer type can be referred to Table 1. Compared to HotNet2, larger accuracies are obtained by CovEx except LAML. However, the sensitivities of CovEx are smaller than those of HotNet2 for a few cancer types. The relatively small number of patients for each cancer type may be the reason why CovEx fails to identify more cancer genes. We also analyzed the genes identified in multiple cancer types. More detailed information can be referred to Supplementary Section 8 and Supplementary Tables 13, 14, 15, 16.

**Table 1:** Comparison results of CovEx and HotNet2 for single cancer types

	NC	SC	AC	NH	SH	AH
BLCA	30	16	53.3%	147	42	28.6%
BRCA	116	53	45.7%	50	18	36.0%
COADREAD	19	13	68.4%	76	19	25.0%
GBM	43	24	55.8%	25	11	44.0%
HNSC	66	30	45.5%	93	23	24.7%
KIRC	76	32	42.1%	23	8	34.8%
LAML	73	38	52.1%	42	28	66.7%
LUAD	52	28	53.8%	240	50	20.8%
LUSC	27	18	66.7%	103	27	26.2%
OV	28	15	53.5%	25	7	28.0%
UCEC	20	15	75.0%	73	27	37.0%

* The first column corresponds to all the analyzed cancer types. For each cancer type, the columns NC, SC, AC and NH, SH, AH correspond to the number of output genes, sensitivities and accuracies of CovEx and HotNet2, respectively.

## DISCUSSION

Cancer genomes exhibit largely different mutation profiles. The long-tail distribution of the frequency cancer mutations arises from tumor heterogeneity. A major challenge in analyzing large-scale genomic profiles of tumor types is to identify the functional driver genes or modules. We presented a novel approach called CovEx for integrative analysis of genomic data of tumors and PPI networks for identifying patient specific driver modules. The approach is based on three basic understandings of driver modules. First, genes in a driver module should be topologically related in a PPI network. Second, most patients should have at least one mutated gene in a driver module (high coverage). Third, most patients have only one mutated gene in that module (high exclusivity). The new approach substantially outperforms some excellent existing approaches. Our approach is based on the following three innovative ideas we developed in this article. (1) CovEx identifies small modules of fixed size *k* belonging to {2, 3, 4, 5} each roots at a node based on Dendrix weight in a constructed influence network which measures the topological relationships between genes in the dataset. Genes in each identified small module would be of close topological relationship. Therefore, these modules are qualified to be the candidates of the oncogenic driver modules to be identified. (2) Although modules of high coverage and exclusivity can be identified in the previous step, some modules with poor exclusivity property may also be identified due to the limitation of the evaluation function. For example, a module of poor exclusivity containing a gene with a significant larger mutation frequency than others may also be identified. We proposed a new exclusivity evaluation metric considering each gene in a module equally which makes up the inherent deficiencies of the previous metric. We further designed a new metric CovEx which can better affect the harmonious property between coverage and exclusivity in the identified modules. We evaluate each module with the new measure and filter those modules with poor exclusivity properties and non-significant CovEx values. Our newly proposed evaluation methodology can be applied to large datasets effectively and efficiently which are superior to the previous ones from the point of view of computational complexity. (3) In the third phase, a minimum set cover model is applied to subsets of patients to identify specific driver modules for each patient. The basic idea comes from the perspective that the solution should satisfy 1) each patient has at least one mutated gene belonging to at least one module to be identified; 2) the number of identified modules is as small as possible. A greedy algorithm was designed to solve the set cover model.

We applied CovEx to three annotated PPI networks, HINT+HI2012, iRefIndex and Multinet for each parameter pairs (λ, *k*), where λ = 0, 1 and *k* = 2, 3, 4, 5. We obtained 24 different solutions each consists of critical modules with 8 on each of the three networks. We proposed a consensus method to extract most possible driver genes and constructed new modules based on all the modules identified by CovEx in each of the 24 single solutions. The consensus method distinguishes all the genes in the 24 single solutions as weight 3, weight 2 and weight 1 genes. All modules in the 24 single solutions are corrected by removing the weight 1 genes. The new consensus modules which are constructed based on the corrected modules exhibited the crosstalk of different functional modules.

In fact, we can also design other consensus methods to extract possible cancer genes or functional modules from different single CovEx predictions. For example, another consensus method was designed by defining a new weight for genes (Supplementary Section 9, Supplementary Tables 17, 18, 19, 20, Supplementary Figure 8). The two consensus methods have comparative performance from the perspective of sensitivities and accuracies of predicted genes. Furthermore, by extracting the common genes predicted by the two consensus methods, we identified a new gene set with much higher accuracy. Comprehensive analysis of the two consensus methods gives us much more understanding of the predicted genes and help to identify the most possible cancer genes or functional modules with fewer false positives (Supplementary Section 10, Supplementary Tables 21, 22, 23, Supplementary Figure 9).

The combinatorial exclusivity evaluation metric CovEx overcomes to some extent the deficiency of other evaluation measures, such as Dendrix weight. Compared to the probabilistic measures, such as mutex, CoMEt, the combinatorial measure is much easier to calculate and enables to identify exclusive modules in a much larger scale. The more comparison results between CovEx and others can be referred to Supplementary Section 11 and Supplementary Figure 10. Results of different CovEx runs demonstrated the stability of CovEx (Supplementary Section 12, Supplementary Table 24).

CovEx is capable of integrating tumor mutation data and PPI networks to reveal infrequent, but functionally important genes and novel functional modules in cancer. We expect that CovEx will be applicable to predict patient-specific driver genes and pathways in future personalized cancer care.

## MATERIALS AND METHODS

### Mathematical model

We denote the mutation data by a matrix *A* = (*a*_ij_) with *m* rows (patients) and *n* columns (tested genes), where *a*_ij_ belongs to {0, 1} indicating whether gene *j* is mutated in patient *i*, i.e. *a*_ij_ = 1 if gene *j* is mutated in patient *i*, and *a*_ij_ = 0 otherwise. For each gene *g*, Γ(*g*) = {*i* : *A*_ig_ = 1} represents the set of patients in which gene *g* is mutated. For a gene set *M*, $\Gamma (M)=\cup_{g\in M}\Gamma (g)$ measures the coverage of *M*. $\omega (M)=\sum_{g\in M}\left|\Gamma (g)\right|-\Gamma (M)$ measures the coverage overlap of *M*. The Dendrix weight introduced by Vandin et al. [17] is employed to discover modules with high coverage and exclusivity:

$$W(M)=\left|\Gamma (M)\right|-\lambda \omega (M)=(1+\lambda )\left|\Gamma (M)\right|-\lambda \sum_{g\in M}\left|\Gamma (g)\right|$$

where λ is a constant parameter controlling the trade-off between coverage and exclusivity of the gene set *M*.

### Influence graph

The PPI networks are integrated to identify topologically related functional modules. The direct topological relationship between genes or proteins can be reflected in the PPI network. However, due to the existence of hub genes and the large difference of gene degrees, uninteresting modules would be identified if we consider the PPI network directly [46]. Based on an annotated PPI network, an insulated heat diffusion process is employed to capture the local topology of the interaction network surrounding a protein (see [25]). An influence matrix *F* is obtained from the process. Elements in the matrix measure the topological relationships between pairs of nodes in the PPI network. Compared to the adjacent matrix, the influence matrix reflects more comprehensive topological relationship information between gene pairs. The influence matrix is not symmetric. A weighted undirected influence graph *G_I_* is defined based on the influence matrix *F* where *F* (*i, j*) is defined as the element of matrix *F* in row *i* and column *j*. The set of nodes of *G_I_* corresponds to the set of tested proteins or their associated genes. The weight of an edge (*g_i_, g_j_*) is defined as *w*(*g*_i_, *g*_j_) = min{*F* (*i, j*), *F* (*j, i*)}. The corresponding genes of the nodes incident with a small weighted edge usually have weak topology relationship. A reduced influence graph *G*_I_(*δ*) is derived by removing all edges with weights smaller than a fixed threshold *δ* [46]. The reduced influence graph can be considered as an approximate PPI network. In our current application, we select *δ* such that the average degree of the nodes in the reduced influence graph is 15 which is approximately the average degree of a real PPI network. For example, the iRefIndex network [47] consists of 91,872 interactions among 12,338 proteins and the MultiNet network [48] consists of 109,597 interactions among 14,445 proteins [25].

### Discovery of network module candidates

The reduced influence graph captures the topological information for each pair of considered genes. The module candidates each roots at a node in the reduced influence graph are calculated by solving a series of binary linear programming (BLP) below.

$$\begin{array}{cc} \max f(x,y)=(1+\lambda )\sum_{i=1}^{m}x_{i}-\lambda \sum_{j=1}^{n}\left(y_{j}\sum_{i=1}^{m}a_{\text{ij}}\right) & \\ \{\begin{array}{c} \sum_{j=1}^{n}a_{ij}y_{j}\geq x_{i},i=1,\cdots ,m\\ \sum_{j=1,\cdots ,n;j\neq v}y_{j}<=k-1\\ x_{i}\in \{0,1\},i=1,\cdots ,m\\ y_{j}\in \{0,1\},j=1,\cdots ,n;j\neq v\\ y_{v}=1 \end{array} & \text{where} \end{array}$$

*m, n* represent the numbers of patients and mutated genes, respectively; *k* is an upper bound of the size of the to-be-identified candidate module; *x_i_* indicates whether some gene in the candidate module is mutated in patient *i*, and *y_j_* indicates whether the candidate module contains gene *j*; *a_ij_* indicates whether gene *j* is mutated in patient *i, i.e*. *a_ij_* = 1 if gene *j* is mutated in patient *i*, and *a_ij_* = 0 otherwise. λ is a parameter pre-specified by users. The first constraint ensures that if genes in the candidate modules are not mutated in patient *i*, then *x_i_* = 0; otherwise *x_i_* = 1.

The BLP problem is NP hard [18]. To solve the BLP efficiently and identify topologically related gene modules, the BLP is restricted to local networks each roots at a node. The local network rooted at a node is extracted by breadth-first search, starting at the node and gradually exploring neighbor nodes until reaching the proper radius or the maximum size which is less than a specified number. The pre-specified number controlling the size of each extracted local network is set to 300 for the dataset in our real application. In our procedure, we first consider λ = 0 which means that the gene set with maximum coverage will be identified. We also consider λ = 1 which means that the exclusive property is considered for identification of gene sets. For both λ = 0 and λ = 1, *k* = 2, 3, 4, 5 are taken to generate candidates, respectively. Other values of λ and *k* can be tested as well. However, the exclusivity property of a module usually tends to get worse as the module size *k* increases. This BLP model is solved by employing the open source software gurobi 6.5.0 (http://www.gurobi.com/).

### Filtration of candidate modules

**Figure 3:**
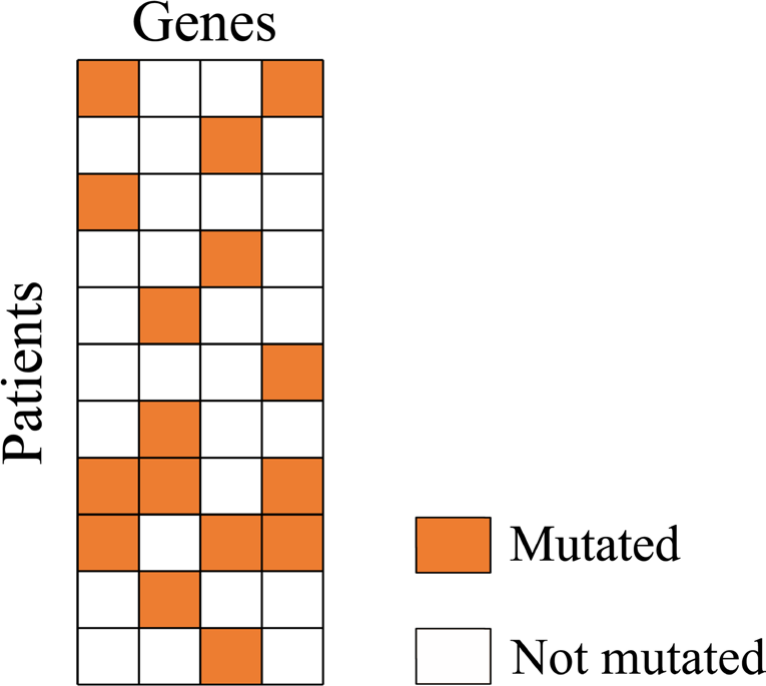
A candidate module mutation matrix In the matrix, 8 patients which have exactly one mutated gene are module exclusive patients. To calculate the exclusivity value Ex of the module, we first calculate the ratio between number of mutated module exclusive patients and number of all mutated patients for each gene. We get 1/4 = 0.25 for the first and the fourth gene, 3/4 = 0.75 for the second and the third gene. The exclusivity value Ex of the module is (0.25 + 0.75 + 0.75 + 0.25)/4 = 0.5. While the coverage value Cov of the module is 11/11 = 1, the CovEx value of the module is 1*0.5 = 0.5.

A few genes with high mutation frequency may dominate the value of the Dendrix weight. Some identified modules contain genes which are not exclusive to the rest. We define a new metric which can coordinate very well between coverage and exclusivity. To do so, we introduce some new terminologies. Patients with only one mutated gene in a module are referred to as module exclusive patients. For each gene in a candidate module, we calculate the ratio between the number of the module exclusive patients mutated in that gene and the number of all patients mutated in that gene. The exclusivity metric for a module, denoted as Ex, is defined as the ratio on average of genes in that module. Modules with exclusivity value Ex less than 0.8 are filtered in our experiment. Biologically, a driver module should not only exhibit higher exclusivity but also larger coverage. The coverage metric for a module, Cov, is defined as the ratio between the number of patients mutated in the module and the number of all patients in the dataset. Then the new metric of a module, denoted by CovEx, is defined as the product of the Cov and Ex of the module (see description of Figure 3). Obviously, the bigger the CovEx of a module, the more significant the module is. We randomly select 100,000 connected sub-networks of size *k* = 2, 3, 4, 5, respectively, in the reduced influence graph and calculate their respective CovEx values. We then obtain 4 distributions of CovEx values for different values of *k* (= 2, 3, 4, 5). We select those significant candidate modules of size *k* by removing from the candidates all those with their *p*-value greater than 0.05 based on the CovEx value distribution for the value of *k*.

### Discovery of crucial candidate modules

To identify the most possible driver module for each specific patient, a minimum set cover model is applied to the set of previous significant candidate modules. The minimum set cover problem can be described as follows:

Given a set I of elements and a collection S of sets whose union equals the set I, it is required to find a smallest subset of S whose union equals the set I.

In our situation, the set *I* is the set of patients covered by all the significant modules, and *S* the collection of patient sets which are 1–1 corresponding to candidate modules obtained after the filtration step, where each patient set in *S* is exactly covered by a module obtained after the filtration step.

The set cover problem is also NP hard. A greedy strategy is adopted to discover the crucial candidate modules. The pseudocode is shown in Table 2.

**Table 2:** Pseudocode of the algorithm for discovering crucial candidate modules

***Input***: a set *I* of patients, all the significant modules (gene sets).
***Output***: a set *C* of modules such that each patient *P*_i_ has mutated genes in *C*.
* A patient is said to be covered by the current *C* if the patient has mutated genes in a module of *C*.*
***Initiation Step***: Set *C ← Ø*.
***While*** some patient has not been covered by the current *C,* ***do***
Choose a module *M* such that the current *C* and *M* cover the largest number of patients in *I*.
Reset *C = C* + *M*.
Return *C*.

The greedy nature of the algorithm implies that the latter a module is selected, the fewer it covers previously uncovered patients. Let *M* be a module output by the above greedy algorithm, *C* (*C’*) a set of modules obtained by the greedy algorithm right before (after) *M* is added to *C*, and *s* a positive integer. If the number of patients covered by *C*’ but not by *C* is no more than *s*, the module *M* is called as an *s*-module.

### Consensus method

For a given mutation dataset, after applying the CovEx to the three annotated PPI networks, HINT+HI2012, iRefIndex and Multinet for each parameter pairs (λ, *k*), where λ = 0, 1 and *k* = 2, 3, 4, 5, we obtained 24 solutions each consists of critical possible driver modules output by CovEx with 8 on each of the three networks. To elucidate the crosstalk of different modules and also refine the identified modules, we then modified a consensus method from [25] to construct consensus networks. To do so, for each of the three PPI networks we assigned to each gene pair a number 1 if the pair belongs to a module of any of the 8 solutions to the PPI network, and 0 otherwise. Doing so, each pair of genes is associated with three numbers of values 0 or 1 according to the three PPI networks, respectively. We then create a double weighted network *N* with genes representing nodes and pairs of genes representing edges with their weights defined to be sum of the three numbers associated with the corresponding pairs of genes. The weight of a node (gene) in *N* is defined to be the maximum weight of the edges incident to the node. All the edges of weight 3 in *N* induce a sub-network with its each component being called a core. Then the final network modules can be obtained by the two steps below:

Step 1. Extend each core by adding all the edges of weight 2, with one end in the core and the other outside the core. The modules obtained in this step are said to be of type 1 in distinguishing from modules of type 2 obtained in Step 2. Especially, if a node of weight 2 connects multiple cores, its corresponding gene which is called a linker gene belongs to all the corresponding type 1 modules.

Step 2. By *N’* we denote the network obtained from *N* by removing all the weight 3 nodes. Then all the network modules of type 2 are those sub-networks induced by all the edges of weight 2 in *N’*.

## SUPPLEMENTARY MATERIALS FIGURES AND TABLES








