# Supplementary material for: Identification of driver modules in pan-cancer via coordinating coverage and exclusivity
Source: Oncotarget. 2017 Mar 21;8(22):36115–26. doi: 10.18632/oncotarget.16433 (PMC5482642; doi:10.18632/oncotarget.16433)
Supplement: Supplementary file 1 [file oncotarget-08-36115-s001.pdf]

Identification of driver modules in pan-cancer via coordinating coverage and exclusivity

Supplementary Materials

SUPPLEMENTARY MATERIALS AND METHODS

If no otherwise specified, the pan-cancer dataset A and the NCG cancer gene list are the dataset and the cancer gene benchmark respectively in the following

Numbers of modules of the 24 solutions

We identified thousands of significant modules in the second phase of CovEx. However, only dozens of modules were identified to be patient oriented driver modules in the third phase. The numbers of identified modules for the 24 single parameter solutions can be referred to Supplementary Tables 1, 2.

Sensitivities and accuracies of the 24 solutions

In the third phase of CovEx, the greedy algorithm selects modules from the previously identified significant modules one by one with the number of new covered patients getting smaller. We further analyzed the solutions of CovEx with *s*-modules filtered for $s = 1, 2, 3$, respectively. The accuracies increased as *s* gets larger for all the 24 solutions. The results can be referred to Supplementary Tables 3–6 and to Supplementary Figure 1.

For each specified value of *s*, we first considered the 8 solutions for each of the three PPI network HINT+HI2012, iRefIndex and Multinet, orderly. For $s = 1$, the minimum sensitivities and the corresponding accuracies were 75, 86, 77 and 57.3%, 51.2%, 59.2%, respectively. The maximum sensitivities and the corresponding accuracies were 97, 116, 109 and 46.6%, 52.0%, 52.7%, respectively. For $s = 2$, the minimum sensitivities and the corresponding accuracies were 63, 75, 70 and 63.6%, 56.4%, 71.4%, respectively. The maximum sensitivities and the corresponding accuracies were 85, 103, 100 and 49.4%, 54.2%, 55.6%, respectively. For $s = 3$, the minimum sensitivities and the corresponding accuracies were 56, 64, 64 and 64.4%, 61.0%, 74.4%, respectively. The maximum sensitivities and the corresponding accuracies were 78, 95, 86 and 53.8%, 55.6%, 57.7%, respectively. The accuracies increase as *s* gets larger for each of the combined solutions of the three PPI networks.

We then considered the 24 solutions for the three PPI networks together. For $s = 1, 2, 3$, the minimum accuracies were 46.6%, 49.4%, 52.9%, and the maximum accuracies were 60.9%, 71.4%, 74.4%, respectively. The accuracies increase as *s* gets larger for the combined solutions of all the three PPI networks.

Furthermore, for $s = 1, 2, 3$, the accuracies of results combining all the 24 solutions were 28.7%, 31.4%, 34.1%, respectively. We observed that the accuracies of the combined results were smaller than each of the solutions. The analysis demonstrated the consistency of the cancer genes and the inconsistency of the false positives in different solutions.

Comparison of CovEx to HotNet2 in single parameter solutions

Based on the mutation frequency score and the optimized parameters, the modules identified by HotNet2 covered 330, 315, 253 genes with 93, 60 and 52 cancer genes of the accuracies 28.2%, 19.0% and 20.6% on HINT+HI2012, iRefIndex and Multinet, respectively [1]. After combination of the three solutions on the three PPI networks, 711 genes with 150 cancer genes were identified. The accuracy was only 21.1%.

For HINT+HI2012, CovEx output 185 genes with 93 cancer genes for $\lambda = 0$ and $k = 2$, reaching its accuracy of 50.3%. For iRefIndex, CovEx output 116 genes with 75 cancer genes for $\lambda = 1$ and $k = 2$ when $s = 3$, reaching its accuracy of 64.7%. For MultiNet, CovEx output 86 genes with 64 cancer genes for $\lambda = 0$ and $k = 2$ when $s = 3$, reaching its accuracy of 74.4% (can be referred to Supplementary Tables 3–5). Obviously, the accuracies of CovEx were much higher than HotNet2, a most popular tool of the same kind, with even larger sensitivities.

The advantage of CovEx over HotNet2

(1) For a given PPI network, there is a containment relationship between the results output by HotNet2 under different parameters. As comparison, the inconsistency of the solutions under different parameters is a unique feature of CovEx. While the accuracies of different solutions are similar, the false positives are largely inconsistent. It is possible to extract more reliable cancer modules or genes from these solutions.

(2) CovEx can provide a hierarchical analysis by filtering *s*-modules from the solutions for different *s*. The hierarchical analysis can provide more accurate predictions and give us more comprehensive understandings of the identified functional modules. This is another unique feature of CovEx over HotNet2.

(3) CovEx can identify topologically related functional modules with significant coverage and exclusivity properties. However, the exclusivity property of the modules identified by HotNet2 could not be guaranteed. CovEx can even identify the possible driver modules for each patient.

Networks results of the consensus method

The consensus networks can be referred to Supplementary Tables 7, 8. All the consensus modules were compared with KEGG pathway and GO database in STRING v10 [2]. For all the enrichment analysis, the statistical background was set to be whole genome in STRING v10. The functional enrichment analysis identified the critical pathways and GO terms related to cancer. The top significantly enriched KEGG pathways and different GO-terms can be referred to Supplementary Table 9. The minimum required interaction score is 0.400. Nodes represent all the proteins produced by a single, protein-coding gene locus. Edges exist if the corresponding proteins jointly contribute to a shared function. It is not necessary that they are physically binding each other. Meanings of edges with different colors can be referred to STRING v10. Especially, the network images of the four largest type 1 sub-networks made in STRING v10 can be referred to Supplementary Figures 2–5.

The largest type 1 sub-network (network index 1, Supplementary Table 7) covers many genes. For the sub-network, while the expected number of edges is 347, the actual number of edges is 955 according to STRING v10. The clustering coefficient is 0.603 and PPI enrichment *p*-value is 0. We further obtained interesting results by comparing the genes in the sub-network to the 138 genes identified by HotNet2 [1]. The common genes identified by CovEx and HotNet2 which belong to at least one of the three cancer gene lists of NCG, CGC or 20/20 rule include ARID1A, ARID2, ATM, CCND1, CCNE1, CDKN2A, EGFR, ERBB2, ERBB4, FBXW7, KDM6A, KRAS, MAP3K1, MDM4, MLL2, MLL3, NRAS, PBRM1, PIK3CA, PIK3R1, SMARCA4, BAP1, BRAF, HRAS, MAP2K4, SMC1A, STK11 and WT1. Seven genes identified by CovEx and HotNet2 do not belong to each of the three cancer gene lists, such as IFT140, ADNP, ANKRD17, CELSR3, PLCE1, TRIP12 and WAPAL. Among them, IFT140, ADNP, ANKRD17, CELSR3, PLCE1, TRIP12 are identified by our two consensus methods (Consensus method A and B). Especially, ANKRD17 was identified by our two consensus methods even with all 7-modules filtered. We believe these genes should be given sufficient attention in the experimental verification of the future. Furthermore, some cancer genes which belong to at least one of the three cancer gene lists were identified by CovEx only. These genes include ABL1, AKT1, APC, CDK4, CREBBP, CTNNB1, DNMT3A, EZH2, GATA3, MDM2, MLLT4, MTOR, MYH11, RB1, SMC3, VHL, WHSC1L1, ZC3HC1, AKAP13, AXIN1, BCL11A, CCND3, CDK6, CDKN1B, CEP170, CHD4, CTCF, DNMT1, FLT3, KDM5A, KIAA1549, MLL4, MYH9, NCOR1, NRXN2, PIK3CG, PLCG1, RPGR, SF3B1, TCF3, USP9X, XPO1 and ZNF217.

Comparison of our consensus method to HotNet2 consensus method

The HotNet2 consensus method outputs all the weight 3 genes and part of weight 2 or weight 1 genes. However, our consensus method outputs all the weight 3 and weight 2 genes. The weight 2 and weight 1 genes selected by HotNet2 consensus method are called as HotNet2 weight 2 genes and HotNet2 weight 1 genes, respectively.

We applied both consensus methods to analyze the 24 CovEx solutions. The accuracies of all the weight 3, weight 2 and weight 1 genes were 72.6%, 43.6%, and 18.8%, respectively. Furthermore, the accuracies of HotNet2 weight 2, weight 1 genes were 46.6% and 20.0%, respectively. Significant differences of the accuracy were observed among different weight genes. The accuracy of the weight 3 genes was high and the accuracy of the weight 1 genes was low. However, the accuracies of the weight 2 and weight 1 genes selected by HotNet2 were barely higher than those of all the weight 2 and weight 1 genes, respectively.

The consensus methods were also applied to the 24 CovEx solutions with all the s-modules filtered for s = 1, 2, 3, respectively. We had similar observations in all the situations. For $s = 1$, the accuracies of all the weight 3, weight 2 and weight 1 genes in the consensus modules were 76.2%, 45.9% and 21.3%, respectively. The corresponding accuracies of HotNet2 weight 2, weight 1 genes were 49.4% and 19.6%, respectively. For $s = 2$, the accuracies of all the weight 3, weight 2 and weight 1 genes were 78.4%, 54.5% and 23.1%, respectively. The corresponding accuracies of HotNet2 weight 2, weight 1 genes were 57.5% and 21.0%, respectively. For $s = 3$, the accuracies of all the weight 3, weight 2 and weight 1 genes were 79.2%, 56.5% and 26.0%, respectively. The corresponding accuracies of HotNet2 weight 2, weight 1 genes were 62.3% and 23.7%, respectively. We also observed that the accuracies of genes with the same weight increased accordingly as s increased.

For CGC and 20/20 rule cancer gene benchmarks, we had the same observations. The sensitivities and accuracies of the solutions obtained based on each of the three cancer gene lists can be referred to Supplementary Tables 10–12. The comparison results can be further referred to Supplementary Figure 6.

Consensus solutions of the three pan-cancer datasets

For each dataset, the consensus method was applied to the 24 solutions obtained for three PPI networks with $\lambda = 0, 1$; $k = 2, 3, 4, 5$, respectively. For pan-cancer dataset A, the consensus method identified 236 genes with 124 cancer genes. The accuracy was 52.5%. When s-modules

were filtered from each solution, the consensus method provided a hierarchical prediction for different values of s. To be specific, the consensus method predicted 196, 163, 140, 126, 116, 98 and 88 genes for $s = 1, 2, 3, 4, 5, 6, 7$, respectively. The corresponding sensitivities and accuracies were 109, 101, 90, 82, 80, 71, 66 and 55.6%, 62.0%, 64.3%, 65.1%, 69.0%, 72.4%, 75%, respectively. Of the 138 genes identified by HotNet2 consensus method in [1], 60 genes were cancer genes. The accuracy was only 43.5%.

For pan-cancer dataset B, the consensus method identified 234 genes with 113 cancer genes. The accuracy was 48.3%. When s-modules were filtered for the single parameter solutions, the consensus method predicted 196, 158, 137, 126, 116, 105 and 101 genes for $s = 1, 2, 3, 4, 5, 6, 7$, respectively. The corresponding sensitivities and accuracies were 105, 91, 86, 81, 76, 71, 69 and 53.6%, 57.6%, 62.8%, 64.3%, 65.5%, 67.6%, 68.3%, respectively. Of the 147 genes identified by HotNet2 consensus method in [1], 54 genes were cancer genes. The accuracy was only 36.7%.

For pan-cancer dataset C, the consensus method identified 261 genes with 122 cancer genes. The accuracy was 46.7%. When s-modules were filtered for the single parameter solutions, the consensus method predicted 213, 155, 129, 118, 108, 100 and 96 genes for $s = 1, 2, 3, 4, 5, 6, 7$, respectively. The corresponding sensitivities and accuracies were 111, 91, 83, 77, 74, 71, 69 and 52.1%, 58.7%, 64.3%, 65.3%, 68.5%, 71.0%, 71.9%, respectively. Of the 99 genes identified by HotNet2 consensus method in [1], 45 genes were cancer genes. The accuracy was only 45.5%.

We observed that CovEx showed great advantage over HotNet2 in both sensitivities and accuracies for all the three pan-cancer datasets. When CGC and 20/20 rule cancer gene lists were selected as comparison benchmarks, we had the same observations. The comparison results of CovEx and HotNet2 for the three pan-cancer datasets can be referred to Supplementary Figure 7.

Results for single cancer types

We analyzed the datasets for all the single cancer types for pan-cancer dataset A. The network results of CovEx for each cancer type can be referred to Supplementary Table 13. More sensitivities and accuracies comparison results between CovEx and HotNet2 can be referred to Supplementary Tables 14, 15. Compared to HotNet2, larger accuracies and competitive sensitivities were obtained by CovEx.

Applying CovEx to each cancer type, we totally obtained 399 genes in which 75 were from at least two cancer types, and 32 from at least three cancer types. Especially, TP53 was identified in all the cancer types, and RB1 in 7 cancer types. Another four genes KRAS, MLL3, MYC and PIK3CA were identified in 6 cancer types. We also observed that the genes identified in multiple cancer types tend to be identified in the pan-cancer dataset. SPEN was the only gene identified in at least three cancer types, but not identified in the pan-cancer dataset. Another interesting observation was that the genes identified in multiple cancer types tend to be identified as weight 3 genes and therefore more attention should be given for the weight 3 genes identified by CovEx in the pan-cancer dataset. All the genes identified by CovEx for at least two cancer types are listed in Supplementary Table 16.

Consensus method B

A new consensus method, denoted as method B, was presented in this section. The method revised from HotNet2 consensus method was denoted as method A.

If a gene pair was contained in some module of a single parameter solution, then the weight of the gene pair was defined as 1 for the solution, and 0 otherwise. For multiple solutions, the weight of a gene pair was defined as the sum of the weight for the solutions. We create the double weighted network N with genes representing nodes and gene pairs representing edges with their weights defined to be the weights of corresponding gene pairs. The weight of a node (gene) in N is defined to be the maximum weight of the edges incident to the node. We then induce a sub-network in N by all the edges of weight no less than a pre-specified number p. All the components of the sub-network are treated as our final network modules.

In our application, the consensus method was applied to the 24 CovEx solutions. In order to determine a proper value of p, we analyzed genes of different weights for 24 solutions. We calculated the accuracy of cancer genes for different weight genes. We also considered the situation that s-modules were filtered for each solution before the consensus method for $s = 1, 2, 3$, respectively. For pan-cancer dataset A, we observed that the accuracy of genes with weight at least 5 was much larger than that of all considered genes in each situation. The value of p was selected as 5 in our experiment. The results can be referred to Supplementary Tables 17–19 and Supplementary Figure 8.

We obtained 8 sub-networks by consensus method B covering 214 genes with 105 cancer genes, reaching the accuracy of 49.1% (Supplementary Table 20). For $s = 1, 2, 3$, the s-modules were filtered for each solution, we identified 90, 81, 76 cancer genes by consensus method B, reaching the accuracies of 61.6%, 65.9%, 73.1%, respectively. The accuracies of identified genes increased accordingly as s increased.

In fact, we also considered another way of defining the weight of gene pairs. The number of all the modules containing the gene pair in all the considered CovEx solutions was defined as the weight of a gene pair. In this situation, the weight of a gene pair for a single parameter solution may be more than 1. However, we obtained similar consensus results for the two situations.

Comprehensive analysis of different consensus methods

The consensus method A identified 236 genes with 124 cancer genes. The accuracy was 52.5%. The consensus method B identified 214 genes with 105 cancer genes. The accuracy was 49.1%. We compared the results of both consensus methods. We identified 162 common genes with 94 cancer genes. The accuracy was 58.0%. Another 74 genes containing 30 cancer genes were identified by consensus method A only. The accuracy was 40.5%. And, 52 genes containing 11 cancer genes were identified by consensus method B only. The accuracy was 21.2%. Compared to genes identified by only one consensus method, much larger accuracy was obtained for genes identified by both consensus methods. In the meantime, we observed that the accuracy of the weight 3 genes of method A was even higher. For $s = 1, 2, 3, 4, 5, 6, 7$, we had the same observations when s-modules were filtered for each solution before both consensus methods. When CGC and 20/20 rule cancer gene lists were selected as comparison benchmarks, we had the same observations. More comparison results can be referred to Supplementary Table 21–23 and Supplementary Figure 9.

Compared to HotNet2 and MSEA, CovEx shows great advantage. Especially, for different s, CovEx gave a hierarchical analysis. For NCG cancer gene benchmarks, HotNet2 outputs 138 genes with 60 cancer genes in [1], denoted by "60 out of 138" for short. The accuracy was 43.5%. Totally, 46 out of 82 genes were identified by MSEA in [3]. The accuracy was 56.1%. As comparison, 64 out of 81 genes were identified by CovEx for $s = 4$. The accuracy was improved to 79.0%.

For CGC cancer gene benchmarks, 45 out of 138 genes and 40 out of 82 genes were identified by HotNet2 and MSEA, respectively. As comparison, 55 out of 90 genes, 49 out of 72 genes and 45 out of 55 genes were identified by CovEx for $s = 3, 5, 7$, respectively. The accuracy was improved from 32.6%, 48.9%, to 61.1%, 68.1% and even 81.8%.

For 20/20 rule cancer gene benchmarks, 40 out of 138 genes and 30 out of 82 genes are identified by HotNet2 and MSEA, respectively. As comparison, 41 out of 72 genes and 38 out of 55 genes identified by CovEx for $s = 5, 7$, respectively. The accuracy was improved from 29.0%, 36.6% to 56.9% and 69.1%.

Comparison to other approaches

Comparison to Dendrix, MDPFinder and Multi-Dendrix

Dendrix weight was a combinatorial measure evaluating exclusivity property for gene sets [4]. Dendrix [4] and MDPFinder [5] adopted different methods to identify modules with large Dendrix weight. Multi-Dendrix [6] introduced a method to identify multiple large Dendrix weight modules simultaneously. As has been mentioned, the Dendrix weight was often biased towards modules with frequently mutated genes [7]. Considering the spectrum of mutational frequencies for cancer mutation profiles that some genes are mutated frequently and the others seldom rarely, the large Dendrix weight modules usually concentrate on a few genes with significant mutation frequency. The identified large Dendrix weight modules may not be exclusive at all.

The advantage of CovEx over the mentioned methods are as follows. CovEx integrated the PPI networks to identify topological related modules. For each considered gene, CovEx identified large Dendrix weight modules in a local network. Much more candidate modules would be identified. Exclusive modules would be further selected by CovEx based on the newly designed exclusivity evaluation measure.

Comparison to muex, mutex, CoMEt, WeSME and WExT

Some statistical exclusivity evaluation measures were designed. The probabilistic generative model developed by muex [8] took error rates into account and assumed that the chance of being altered for each gene in a module was equal. A statistical test of mutual exclusivity was derived by comparing its likelihood to the null model that assumed independent gene alterations. The muex score was still sensitive to high frequency mutations [7]. Another measure mutex [9] tested each gene against the union of all other alterations in the module, and obtained the least significant p value which was further corrected for multiple hypothesis testing. Mutex ensured that each gene in the module significantly contributed to the mutual exclusivity pattern. A greedy algorithm was applied to a subregion of a constructed network to search for exclusive modules in [9]. The limitation of the search space was due to the computational complexity for the multiple hypothesis testing. Although pathway crosstalk is common in cancer, neither muex algorithm nor mutex algorithm identifies overlapping gene sets. CoMEt [7] evaluated mutual exclusivity for modules conditional on the observed frequency of each alteration. CoMEt was also less biased towards high frequency alterations. An MCMC algorithm was applied to perform simultaneous identification of multiple mutually exclusive modules. Both WExT [10] and WeSME [11] approximate the random permutation test for exclusivity evaluation where the number of mutations in each event and each sample are fixed.

We expand on these probabilistic approaches by designing a novel combinatorial exclusive metric Ex. The measure Ex ensures that every gene in the module significantly contributes to the pattern. The advantage of CovEx is that the combinatorial measure Ex is effective and much easier to be calculated. Due to the reduction of the computational complexity, CovEx is applicable for exhaustive search for large datasets. CovEx searches for exclusive modules for each considered gene in a local constructed influence network. Furthermore, the

independence of the identification of modules would result in the possible overlap of different modules. This helps to further study the crosstalk of identified modules.

Comparison to MEMo, MEMCover and MUFFINN

MEMo [12] and MEMCover [13] were both network based exclusive modules identification methods. Both methods employed the random permutation test method to evaluate exclusivity of modules. Furthermore, both methods restricted the analysis to gene sets that interact in a PPI network. MEMo failed to run for large dataset. MEMCover only utilized pairwise mutual exclusivity information to identify modules. The genes in the module may not be completely exclusive. Different from MEMCover that greedily selected genes and created modules according to the PPI edge confidence score and mutual exclusive score for edges in the PPI network, CovEx first identified candidate mutual exclusive modules by maximizing Dendrix score in a local network and further selected significant modules based on a newly designed exclusive measure. Furthermore, CovEx selected the patient-specific driver modules by a minimum set cover model.

The advantage of CovEx over the mentioned methods are as follows. (1) The exclusivity of the identified module was evaluated by Ex. Genes in the identified module must be mutually exclusive. (2) The genes in the identified modules were topologically related, but not necessary to interact in the PPI network. More de novo discoveries may be possible for CovEx due to the incomplete knowledge of the current pathway and PPI networks. (3) Compared to the computing expensive random permutation test method, the computational complexity of the combinatorial evaluation method was much lower.

We also compared the results of MEMCover to CovEx. MEMCover identified 879 genes orderly based on HumanNet [13]. We further considered MUFFINN [14], a cancer gene prioritizing method, to make comparison. MUFFINN presented two different scoring methods, NDmax and NDsum, based on two different PPI networks, Humannet and String v10. We run MUFFINN for the pan-cancer dataset A. To compare with CovEx consensus results, we selected the top 236, 196, 163, 140, 126, 116, 98 and 88 genes of MEMCover and MUFFINN which corresponded to numbers of CovEx consensus genes for $s = 0, 1, \ldots\ldots, 7$, respectively. The comparison results can be referred to Supplementary Figure 10. For different cancer gene benchmarks, the comparison results were incompletely consistent. Still, we observed significant advantage of CovEx over MEMCover and MUFFINN. The comparison results demonstrated the good performance of CovEx.

Results of different CovEx runs

We run CovEx for pan-cancer dataset A for another two runs. Only small differences were observed between different runs. The results demonstrated the stability of CovEx. The comparison results can be referred to Supplementary Table 24.

REFERENCES

1. Leiserson MD, Vandin F, Wu HT, Dobson JR, Eldridge JV, Thomas JL, Papoutsaki A, Kim Y, Niu B, McLellan M, Lawrence MS, Gonzalez-Perez A, Tamborero D, et al. Pan-cancer network analysis identifies combinations of rare somatic mutations across pathways and protein complexes. Nature genetics. 2015; 47:106–114.
2. Szklarczyk D, Franceschini A, Wyder S, Forslund K, Heller D, Huerta-Cepas J, Simonovic M, Roth A, Santos A, Tsafou KP, Kuhn M, Bork P, Jensen LJ, et al. STRING v10: protein-protein interaction networks, integrated over the tree of life. Nucleic acids research. 2015; 43:D447–452.
3. Jia PL, Wang Q, Chen QX, Hutchinson KE, Pao W, Zhao ZM. MSEA: detection and quantification of mutation hotspots through mutation set enrichment analysis. Genome Biol. 2014; 15.
4. Vandin F, Upfal E, Raphael BJ. De novo discovery of mutated driver pathways in cancer. Genome research. 2012; 22:375–385.
5. Zhao J, Zhang S, Wu LY, Zhang XS. Efficient methods for identifying mutated driver pathways in cancer. Bioinformatics. 2012; 28:2940–2947.
6. Leiserson MD, Blokh D, Sharan R, Raphael BJ. Simultaneous identification of multiple driver pathways in cancer. Plos Comput Biol. 2013; 9:e1003054.
7. Leiserson MD, Wu HT, Vandin F, Raphael BJ. CoMEt: a statistical approach to identify combinations of mutually exclusive alterations in cancer. Genome Biol. 2015; 16:160.
8. Szczurek E, Beerenwinkel N. Modeling mutual exclusivity of cancer mutations. Plos Comput Biol. 2014; 10:e1003503.
9. Babur O, Gonen M, Aksoy BA, Schultz N, Ciriello G, Sander C, Demir E. Systematic identification of cancer driving signaling pathways based on mutual exclusivity of genomic alterations. Genome Biol. 2015; 16:45.
10. Leiserson MD, Reyna MA, Raphael BJ. A weighted exact test for mutually exclusive mutations in cancer. Bioinformatics. 2016; 32:i736–i745.
11. Kim YA, Madan S, Przytycka TM. WeSME: uncovering mutual exclusivity of cancer drivers and beyond. Bioinformatics. 2016.
12. Ciriello G, Cerami E, Sander C, Schultz N. Mutual exclusivity analysis identifies oncogenic network modules. Genome research. 2012; 22:398–406.
13. Kim YA, Cho DY, Dao P, Przytycka TM. MEMCover: integrated analysis of mutual exclusivity and functional network reveals dysregulated pathways across multiple cancer types. Bioinformatics. 2015; 31:i284–292.
14. Cho A, Shim JE, Kim E, Supek F, Lehner B, Lee I. MUFFINN: cancer gene discovery via network analysis of somatic mutation data. Genome Biol. 2016; 17:129.

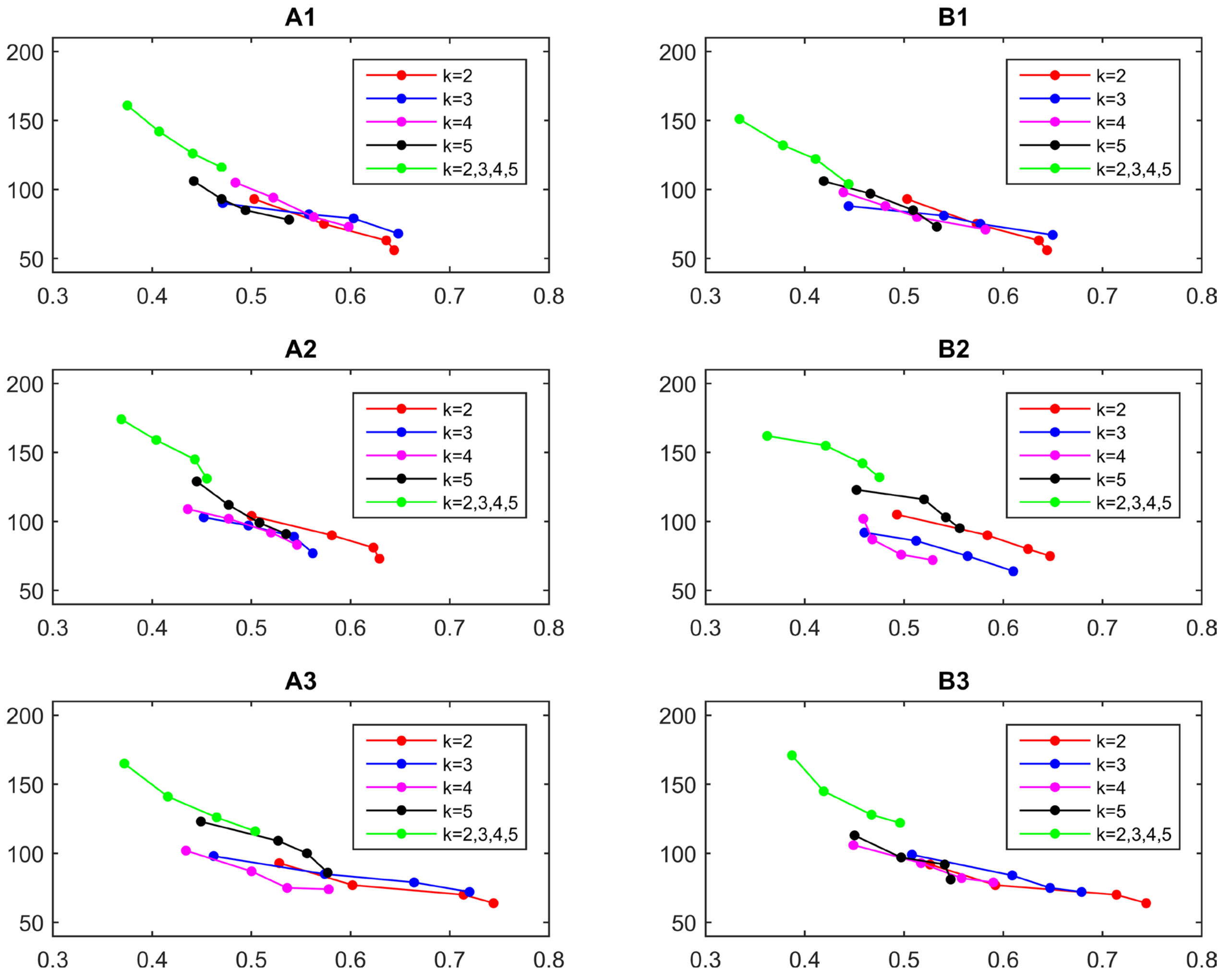

Supplementary Figure 1: The 24 CovEx solutions and some combination results. (**A1**, **B1**) are obtained for HINT + HI2012; (**A2**, **B2)** for iRefIndex and (**A3**, **B3)** for Multinet. The horizontal axis and vertical axis in each panel show the accuracy and sensitivity, respectively. (A1, A2, A3) correspond to $\lambda = 0$; (B1, B2, B3) correspond to $\lambda = 1$. The four points in each polyline in each panel correspond to the solution of CovEx for $s = 0, 1, 2, 3$, respectively. "$k = 2$", "$k = 3$", "$k = 4$", "$k = 5$" correspond to CovEx solutions of corresponding values of k, and "$k = 2, 3, 4, 5$" corresponds to CovEx results combining the four solutions for specific λ and PPI network.

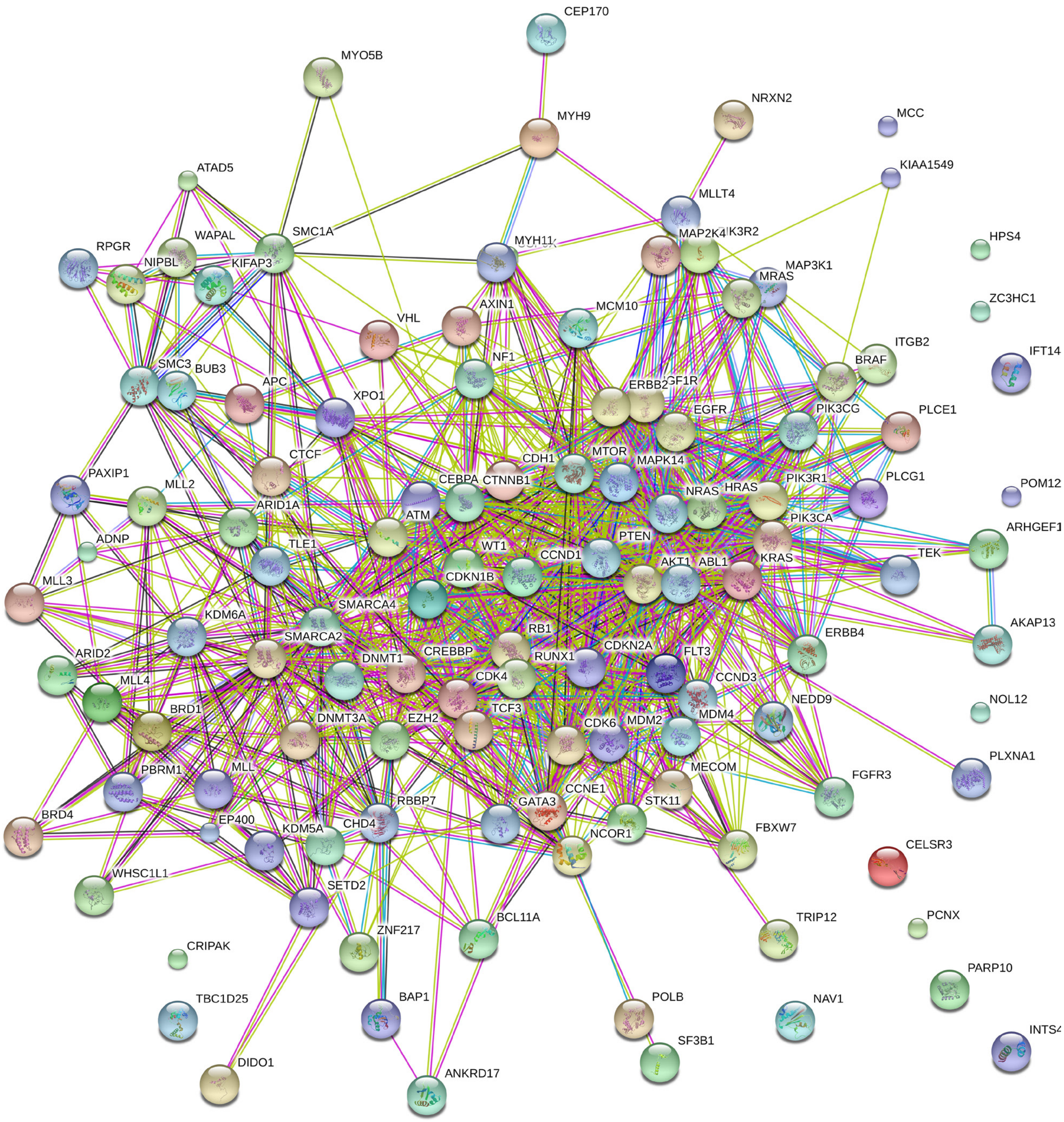

Supplementary Figure 2: Network image for the largest type 1 sub-network (network index 1).

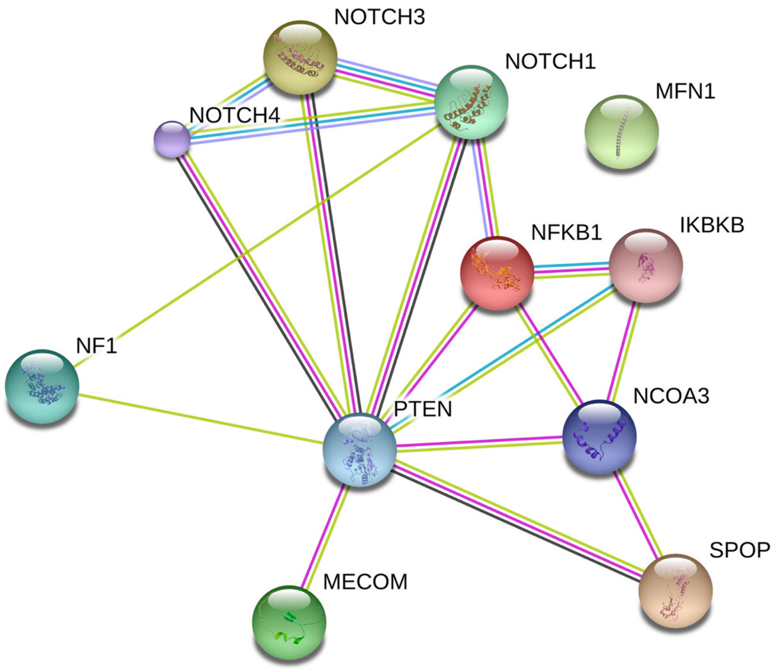

Supplementary Figure 3: Network image for the type 1 sub-network of network index 5.

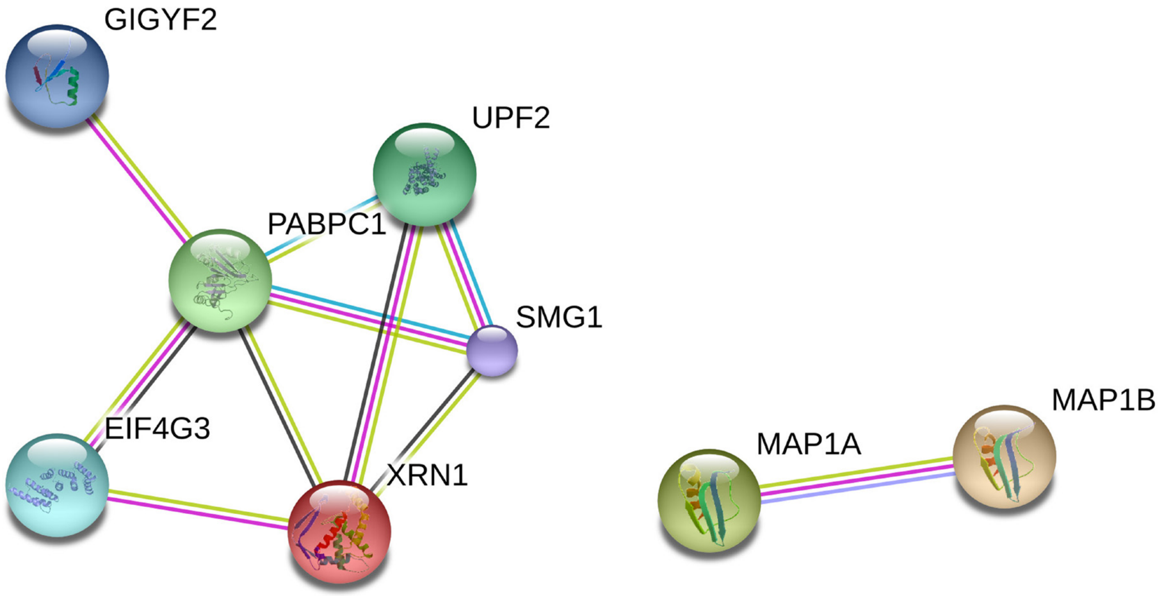

Supplementary Figure 4: Network image for the type 1 sub-network of network index 8.

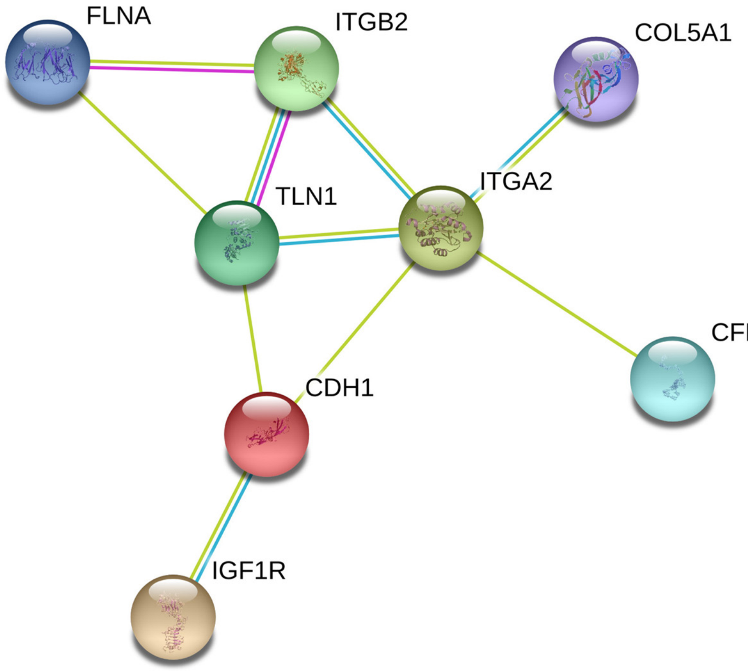

Supplementary Figure 5: Network image for the type 1 sub-network of network index 13.

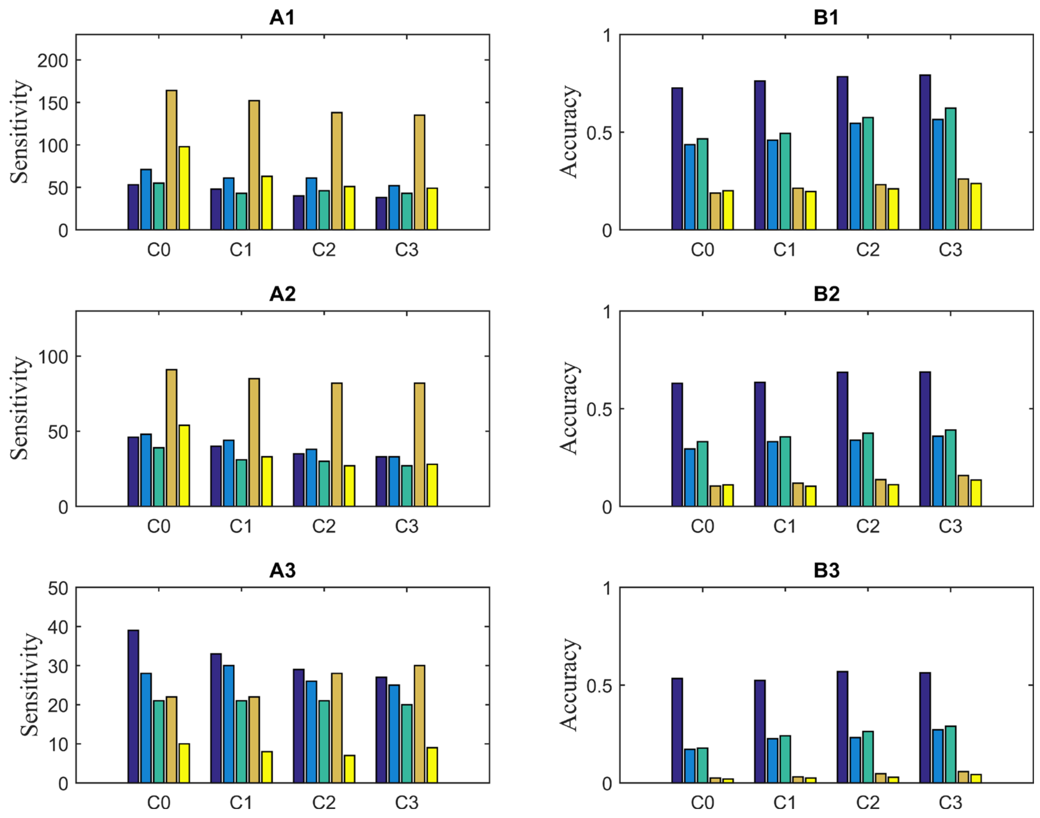

Supplementary Figure 6: Comparison of different weight genes and genes selected by HotNet2 consensus method. The comparison benchmarks for (**A1**, **B1**), **(A2**, **B2)** and (**A3**, **B3)** are NCG, CGC and 20/20 rule cancer gene lists, respectively. C0, C1, C2 and C3 in each panel correspond to the CovEx solutions for $s = 0, 1, 2, 3$, respectively. For each situation, the meanings of the five bars from left to right are weight 3 genes, all weight 2 genes, HotNet2 weight 2 genes, all weight 1 genes and HotNet2 weight 1 genes, respectively.

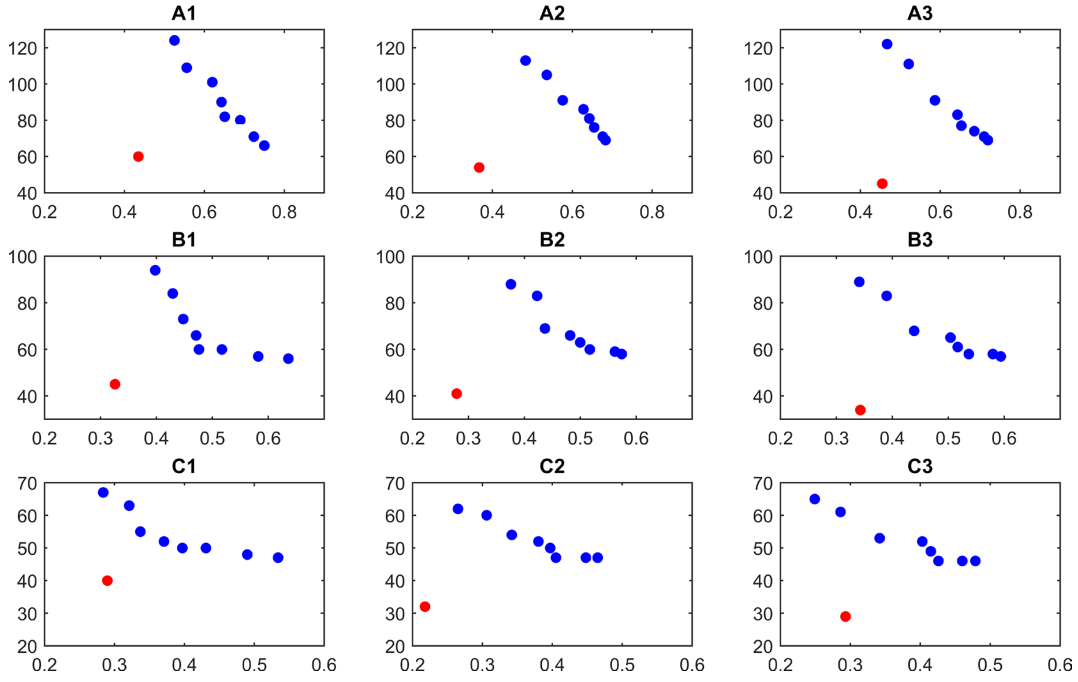

Supplementary Figure 7: Comparison results of CovEx and HotNet2 for the three pan-cancer datasets. The horizontal axis and vertical axis in each panel shows the accuracy and the sensitivity, respectively. The panels (**A1**, **B1**, **C1**) correspond to pan-cancer dataset A, (**A2**, **B2**, **C2**) correspond to dataset B and (**A3**, **B3**, **C3**) correspond to dataset C. The comparison benchmarks for (**A1**, **A2**, **A3**), (**B1**, **B2**, **B3**) and (**C1**, **C2**, **C3**) are NCG, CGC and 20/20 rule cancer gene lists, respectively. The red point in each panel correspond to HotNet2 result and the eight blue points correspond to CovEx consensus results for *s* = 0, 1, 2, 3, 4, 5, 6, 7, respectively. The accuracy of CovEx results increases as *s* increase.

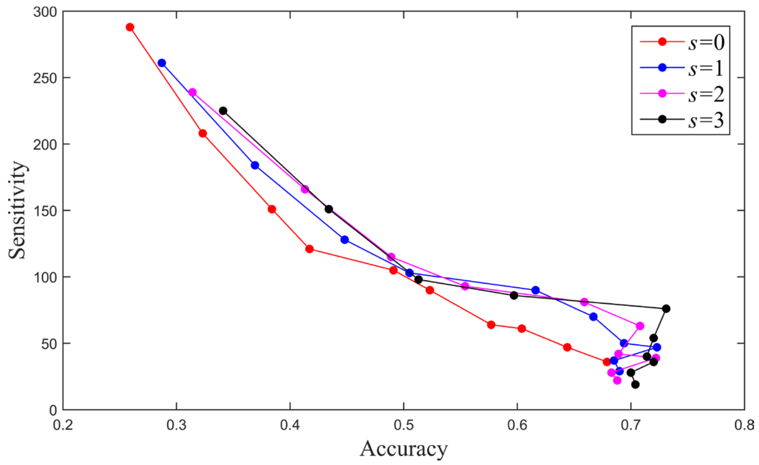

Supplementary Figure 8: Different weight genes for 24 single parameter CovEx solutions. The weight of genes were defined by consensus method B. The four polylines correspond to the CovEx results for $s = 0, 1, 2, 3$, respectively. The 10 points in each polyline correspond to genes with weight at least 1, at least 2, up to at least 10. The accuracy of genes with weight at least 10 is larger than that of genes with weight at least 1 in each of the 4 polylines.

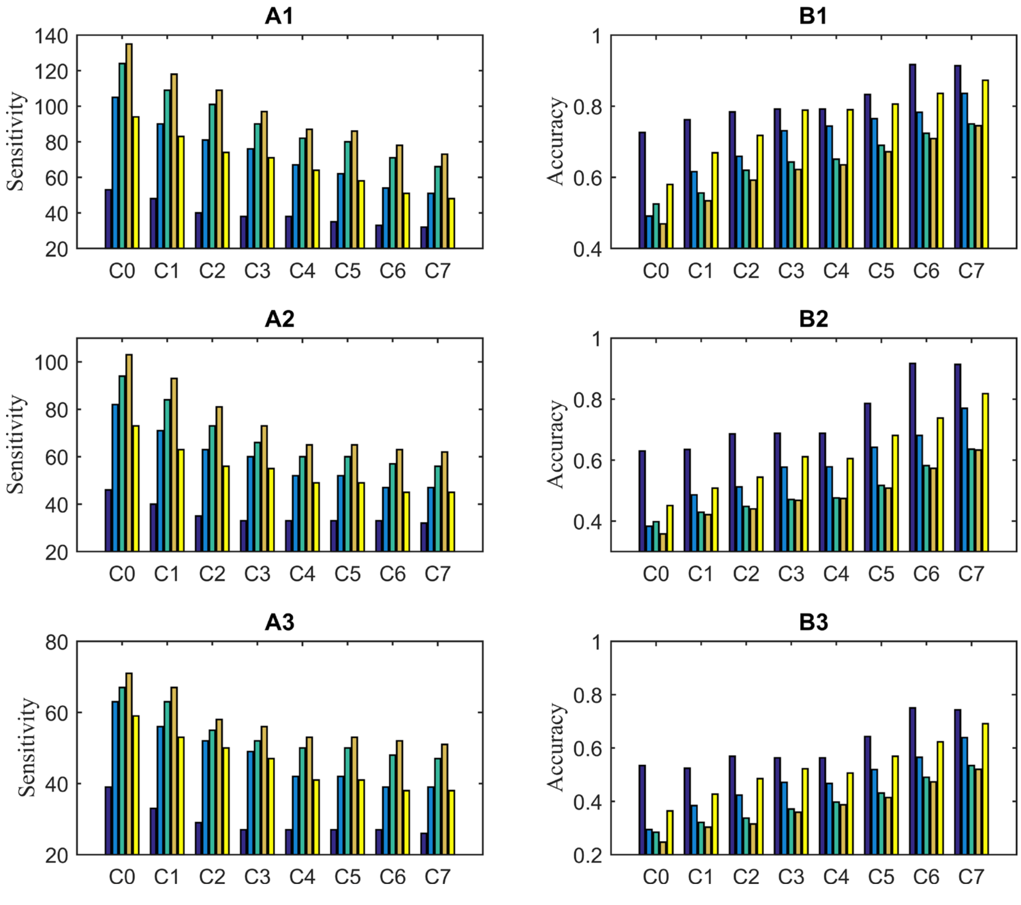

Supplementary Figure 9: Comparison results of genes identified by consensus method A and B. The comparison benchmarks for (**A1**, **B1**), (**A2**, **B2**) and (**A3**, **B3**) are NCG, CGC and 20/20 rule cancer gene lists, respectively. For each situation, the meanings of the five bars from left to right are weight 3 genes, genes of method A, genes of method B, combined genes of both methods and common genes of both methods, respectively.

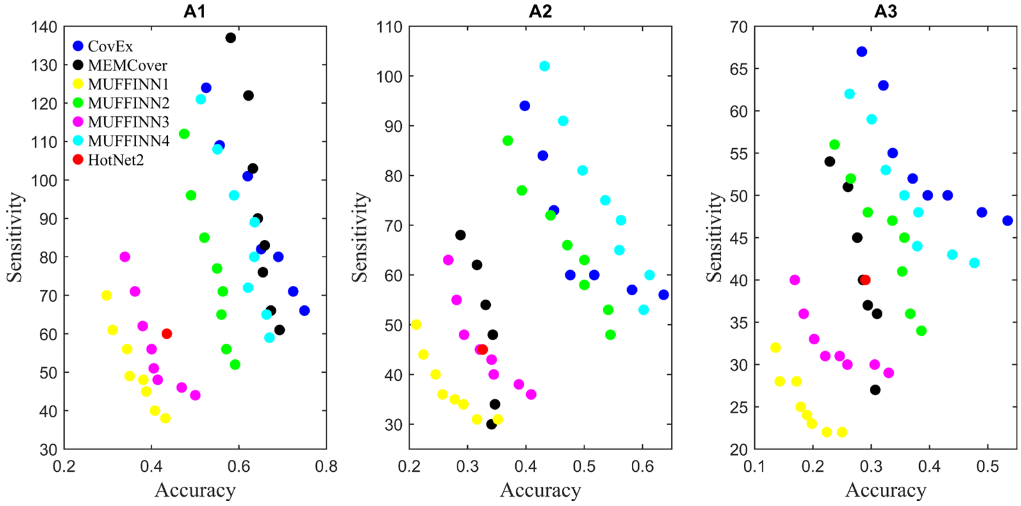

Supplementary Figure 10: Comparison of CovEx to other approaches. The red point in each panel corresponds to HotNet2 result. The eight blue points correspond to CovEx consensus results for $s = 0, 1, 2, 3, 4, 5, 6, 7$, respectively. MUFFINN1, MUFFINN2 correspond to NDmax and NDsum methods based on the PPI network Humannet, respectively. MUFFINN3, MUFFINN4 correspond to NDmax and NDsum methods based on the PPI network String v10, respectively. The cancer gene benchmarks for **A1**, **A2** and **A3** are NCG, CGC and 20/20 rule cancer gene lists, respectively.

Supplementary Table 1: Numbers of identified modules for the 12 CovEx solutions with Dendrix weight parameter λ = 0

λ = 0	*k* = 2	*k* = 3	*k* = 4	*k* = 5
HINT+HI2012	1699 / 114	3709 / 92	3425 / 82	2865 / 75
iRefIndex	2049 / 125	5486 / 102	5488 / 88	5073 / 86
MultiNet	2192 / 110	4540 / 103	4324 / 84	3920 / 78

*For each number pair A / B, A is the number of significant modules identified by CovEx in the second phase, B is the number of patient specific modules identified by CovEx in the third phase.

Supplementary Table 2: Numbers of identified modules for the 12 CovEx predictions with Dendrix weight parameter λ = 1

λ = 1	*k* = 2	*k* = 3	*k* = 4	*k* = 5
HINT+HI2012	1701 / 114	3912 / 93	3685 / 79	3199 / 70
iRefIndex	2050 / 129	5526 / 87	5613 / 76	5387 / 80
MultiNet	2205 / 109	4635 / 99	4491 / 87	4178 / 74

*The number pair A / B has the same meaning as in Supplementary Table 1.

Supplementary Table 3: CovEx solutions for PPI network HINT+HI2012

	k = 2	*k* = 3	*k* = 4	*k* = 5	*k* = 2, 3, 4, 5
λ = 0	93/185/50.3%	90/191/47.1%	105/217/48.4%	106/240/44.2%	161/429/37.5%
	75/131/57.3%	82/147/55.8%	94/180/52.2%	93/198/47.0%	142/349/40.7%
	63/99/63.6%	79/131/60.3%	80/142/56.3%	85/172/49.4%	126/286/44.1%
	56/87/64.4%	68/105/64.8%	73/122/59.8%	78/145/53.8%	116/247/47.0%
λ = 1	93/185/50.3%	88/198/44.4%	98/223/43.9%	106/253/41.9%	151/452/33.4%
	75/131/57.3%	81/150/54.0%	88/183/48.1%	97/208/46.6%	132/349/37.8%
	63/99/63.6%	75/130/57.7%	80/156/51.3%	85/167/50.9%	122/297/41.1%
	56/87/64.4%	67/103/65.0%	71/122/58.2%	73/137/53.3%	104/234/44.4%
λ = 0, λ = 1	93/185/50.3%	98/232/42.2%	125/296/42.2%	126/337/37.4%	176/553/31.8%
	75/131/57.3%	88/171/51.5%	114/250/45.6%	115/283/40.6%	154/442/34.8%
	63/99/63.6%	82/149/55.0%	98/203/48.3%	104/238/43.7%	141/370/38.1%
	56/87/64.4%	71/112/63.4%	88/169/52.1%	95/195/48.7%	130/306/42.5%

*For each parameter pair (λ, *k*), the values A/B/C correspond to the sensitivity, the number of predicted genes and the accuracy of CovEx, and further those for parameter *s* = 1, 2, 3, orderly. Especially, "*k* = 2,3,4,5" corresponds to combined CovEx solutions of the four solutions under corresponding values of *k*, "λ = 0, λ = 1" corresponds to combined solutions of λ = 0 and λ = 1. The cancer gene benchmark is NCG cancer gene list.

Supplementary Table 4: CovEx solutions for PPI network iRefIndex

	k = 2	*k* = 3	*k* = 4	*k* = 5	*k* = 2, 3, 4, 5
λ = 0	104/208/50.0%	103/228/45.2%	109/250/43.6%	129/290/44.5%	174/472/36.9%
	90/155/58.1%	97/195/49.7%	102/214/47.7%	112/235/47.7%	159/394/40.4%
	81/130/62.3%	89/164/54.3%	92/177/52.0%	99/195/50.8%	145/327/44.3%
	73/116/62.9%	77/137/56.2%	83/152/54.6%	91/170/53.5%	131/288/45.5%
λ = 1	105/213/49.3%	92/200/46.0%	102/222/45.9%	123/272/45.2%	162/447/36.2%
	90/154/58.4%	86/168/51.2%	87/186/46.8%	116/223/52.0%	155/368/42.1%
	80/128/62.5%	75/133/56.4%	76/153/49.7%	103/190/54.2%	142/310/45.8%
	75/116/64.7%	64/105/61.0%	72/136/52.9%	95/171/55.6%	132/278/47.5%
λ = 0, λ = 1	109/221/49.3%	116/286/40.6%	127/312/40.7%	154/371/41.5%	200/579/34.5%
	96/167/57.5%	110/243/45.3%	117/275/42.5%	143/317/45.1%	189/497/38.0%
	87/141/61.7%	98/198/49.5%	106/230/46.1%	129/267/48.3%	174/417/41.7%
	79/126/62.7%	90/170/52.9%	96/198/48.5%	120/238/50.4%	163/378/43.1%

*The meanings of values A/B/C in the table are similar to those in Supplementary Table 3.

Supplementary Table 5: CovEx solutions for PPI network MultiNet

	k = 2	*k* = 3	*k* = 4	*k* = 5	*k* = 2, 3, 4, 5
λ = 0	93/176/52.8%	98/212/46.2%	102/235/43.4%	123/274/44.9%	165/443/37.2%
	77/128/60.2%	85/148/57.4%	87/174/50.0%	109/207/52.7%	141/339/41.6%
	70/98/71.4%	79/119/66.4%	75/140/53.6%	100/180/55.6%	126/271/46.5%
	64/86/74.4%	72/100/72.0%	74/128/57.8%	86/149/57.7%	116/230/50.4%
λ = 1	92/175/52.6%	99/195/50.8%	106/236/44.9%	113/251/45.0%	171/442/38.7%
	77/130/59.2%	84/138/60.9%	93/180/51.7%	97/195/49.7%	145/346/41.9%
	70/98/71.4%	75/116/64.7%	82/147/55.8%	92/170/54.1%	128/274/46.7%
	64/86/74.4%	72/106/67.9%	79/134/59.0%	81/148/54.7%	122/246/49.6%
λ = 0, λ = 1	93/178/52.2%	116/262/44.3%	120/291/41.2%	146/368/39.7%	193/557/34.6%
	77/130/59.2%	101/190/53.2%	103/224/46.0%	130/295/44.1%	166/446/37.2%
	70/98/71.4%	86/147/58.5%	94/189/49.7%	119/251/47.4%	148/363/40.8%
	64/86/74.4%	80/129/62.0%	92/172/53.5%	102/208/49.0%	138/314/43.9%

*The meanings of values A/B/C in the table are similar to those in Supplementary Table 3.

Supplementary Table 6: Combined CovEx solutions of all the three PPI networks HINT+HI2012, iRefIndex and MultiNet

	k = 2	*k* = 3	*k* = 4	*k* = 5	*k* = 2, 3, 4, 5
λ = 0	145/336/43.2%	148/412/35.9%	158/448/35.3%	188/533/35.3%	251/857/29.3%
	123/249/49.4%	133/316/42.1%	143/357/40.1%	166/422/39.3%	223/692/32.2%
	107/194/55.2%	122/261/46.7%	128/292/43.8%	149/354/42.1%	200/564/35.4%
	102/176/58.0%	112/216/51.9%	119/258/46.1%	133/299/44.5%	187/490/38.2%
λ = 1	148/344/43.0%	138/383/36.0%	158/439/36.0%	176/511/34.4%	242/867/27.9%
	125/252/49.6%	125/286/43.7%	141/358/39.4%	159/413/38.5%	216/684/31.6%
	111/198/56.1%	108/234/46.2%	124/297/41.8%	144/348/41.4%	200/577/34.7%
	105/178/59.0%	100/191/52.4%	113/251/45.0%	128/305/42.0%	186/501/37.1%
λ = 0, λ = 1	149/349/42.7%	166/514/32.3%	193/593/32.5%	224/718/31.2%	288/1110/25.9%
	127/260/48.8%	150/389/38.6%	175/490/35.7%	204/599/34.1%	261/910/28.7%
	112/204/54.9%	132/316/41.8%	157/406/38.7%	187/507/36.9%	239/761/31.4%
	107/185/57.8%	123/263/46.8%	144/351/41.0%	171/436/39.2%	225/660/34.1%

*The meanings of values A/B/C in the table are similar to those in Supplementary Table 3. The difference is that the values A/B/C are obtained for combination results of all the three mentioned PPI networks for the corresponding parameters λ and *k*.

Supplementary Table 7: Type 1 sub-networks constructed by the consensus method A

Network index	Core network (Weight 3 genes)	Weight 2 genes with weight 3 partners	Linkers
1	ABL1[1](111) AKT1[8](111) APC[8](111) ARID1A[8](111) ARID2[1](111) ATM[8](111) CCND1[8](111) CCNE1[8](110) CDK4[8](110) CDKN2A[8](111) CREBBP[8](111) CTNNB1[4](111) DNMT3A[8](111) EGFR[8](111) ERBB2[8](111) ERBB4[2](100) EZH2[4](111) FBXW7[8](111) GATA3[8](111) IFT140[2] KDM6A[3](111) KRAS[8](111) MAP3K1[8](111) MDM2[8](111) MDM4[2](111) MLL2[8](111) MLL3[8](111) MLLT4[8](110) MTOR[8](100) MYH11[2](110) NRAS[8](111) PBRM1[8](111) PIK3CA[8](111) PIK3R1[8](111) POM121C[2] RB1[8](111) SMARCA2[3] SMARCA4[8](111) SMC3[2](100) VHL[8](111) WHSC1L1[8](110) ZC3HC1[1]	ADNP[1] AKAP13[4](100) ANKRD17[8] ARHGEF1[2] ATAD5[2] AXIN1(111) BAP1[8](111) BCL11A(110) BRAF[2](111) BRD1 BUB3[1] CCND3(110) CDK6(110) CDKN1B[1](110) CELSR3[2] CEP170(100) CHD4[6](100) CRIPAK[4] CTCF[8](100) DIDO1 DNMT1[3](101) EP400[1] FLT3[8](111) HPS4[2] HRAS(111) INTS4[7] KDM5A[2](110) KIAA1549[2](110) KIFAP3 MAP2K4(111) MAPK14[1] MCC[3] MCM10[1] MLL[7] MLL4(100) MRAS MYH9(110) MYO5B[2] NAV1[2] NCOR1[8](111) NEDD9[3] NIPBL NOL12[1] NRXN2[5](100) PARP10[8] PAXIP1 PCNX[1] PIK3CG[1](100) PIK3R2 PLCE1[4] PLCG1(110) PLXNA1[1] POLB RBBP7 RPGR[5](100) SF3B1[1](111) SMC1A[5](100) STK11[1](111) TBC1D25 TCF3[1](110) TEK TLE1 TRIP12[2] USP9X(100) WAPAL WT1[4](111) XPO1[2](110) ZNF217[1]	BRD4[6](110) CDH1[4](111) CEBPA[4](111) FGFR3[8](111) IGF1R[1] ITGB2[1] MECOM[8](110) NF1[6](111) PTEN[8](111) RUNX1[8](111) SETD2[8](111)
2	NPM1[8](111) TP53[8](111)	CENPF HLA-B(100)	CEBPA[4](111)
3	MED12[8](111) MED13[8]	MED14	BRD4[6](110)
4	SPTAN1[6] SPTB[6]		
5	IKBKB[1](010) NCOA3[1](001) NOTCH1[6](111)	MFN1 NFKB1[1] NOTCH3[6](100) NOTCH4 SPOP(111)	MECOM[8](110) NF1[6](111) PTEN[8](111)
6	F8[6](100) PROS1[6]		COL5A1[3](100)
7	LILRB1[5](100) LILRB2[5]	HLA-DRB1[1]	
8	MAP1A[5] PABPC1[8](100) SMG1[6] UPF2[5]	EIF4G3 GIGYF2[7](100) MAP1B[1] XRN1[2]	
9	BCL2L1[8] MCL1[8]	FRYL MYO5A(010)	
10	CHD6[5](100) TLR4[5](100)	SETDB1 TOM1L2	
11	KIT[8](111) PTPN11[8](111)	CBLB(110) LIFR(110)	
12	GOLGB1[2] SREBF2[2]		FGFR3[8](111)
13	ITGA2[1] TLN1[1]	CFH[1] FLNA	CDH1[4](111) COL5A1[3](100) IGF1R[1] ITGB2[1]
14	EP300[8](111) MYC[8](111)		
15	KAT6A[8](110) KAT6B[8](110)	ATN1	RUNX1[8](111) SETD2[8](111)

*The number s in the bracket means that the gene was further identified by consensus method B with (s-1)-modules filtered. The three numbers in the parenthesis indicate whether the gene belongs to NCG, CGC and 20/20 rule cancer gene list, orderly. If the gene belongs to the corresponding cancer gene list, the number is 1, and 0 otherwise.

Supplementary Table 8: Type 2 sub-networks constructed by consensus method A

Network index	Type 2 sub-networks
1	NLRP1(100) NLRP3(100)
2	NPHP4 TNS1[3] TSC22D1(100) WNK1[3]
3	CNOT1 CNOT3(110)
4	CACNA1A HIVEP1[1]
5	ITPR1 ITPR2
6	CUX1(010) MYCL1[3](001)
7	CDH24(100) HLA-A(110)
8	CAD IDH1[6](111)
9	ADRBK1[1] PDGFRB[1](110) PRKCB(100)
10	AKAP13[4](100){1} AKAP3[3] ARHGAP5 CDH1[4](111){1,13} CIT[3] MYO7A RANBP2[1](010)
11	ABCA1 CELSR3[2]{1} EIF4G3{8} GIGYF2[7](100){8} PRPF8 TRIP12[2]{1} UACA XPO1[2](110){1}
12	BACH1 BACH2 CEBPA[4](111){1,2} NCOR1[8](111){1} NFE2L2(111) POLB{1} RUNX1[8](111){1,15} TLE1{1}
13	CHD4[6](100){1} DHX33[1] RBBP7{1} SHPRH[3] USP36[3]
14	CTCF[8](100){1} FGFR2(111) FGFR3[8](111){1,12}
15	KIAA1549[2](110){1} MYO5B[2]{1}
16	C3[1](100) CFH[1]{13} CR1[1]
17	RPGR[5](100){1} SMC1A[5](100){1} STAG2(111)
18	ATN1{15} IGF1R[1]{1,13} MAGI2 PTEN[8](111){1,5} STK11[1](111){1} ZNF91
19	GLUD2 NAV1[2]{1} TET2[2](111)
20	HPS4[2]{1} MYH9(110){1}
21	INTS4[7]{1} POLR2A[8] PPP2R1A[4](111) SETD2[8](111){1,15} SUPT6H[8] ZNF687[7](100)
22	ADAMTS1[1] ANKRD17[8]{1} ASXL1(111) BAP1[8](111){1} FLT1[1](100) HCFC1[1] NIN(110) RB1CC1 RBMX[7](100) SRRM2[7]
23	NOTCH3[6](100) NOTCH4
24	MLL[7]{1} SBF1
25	EED(100) KDM5A[2](110){1}
26	BPTF(100) MECOM[8](110){1,5}
27	DNMT1[3](101){1} SUZ12(110) WT1[4](111){1}
28	BRD1{1} TBC1D25{1}
29	ATAD5[2]{1} BRD4[6](110){1,3} PDGFRA[1](111)
30	PCNX[1]{1} ZNF217[1]{1}
31	FRYL{9} NUP160
32	ARHGEF1[2]{1} HRAS(111){1}

*Numbers in the bracket or parenthesis have the same meanings as in Supplementary Table 7. The number in the brace indicates that the gene also belongs to the type 1 network with the corresponding network index.

Supplementary Table 9: The top enriched functional pathways or GO terms for some type 1 sub-networks constructed by consensus method A. See Supplementary_Table_9

Supplementary Table 10: Different weight genes of the 24 CovEx solutions with comparison benchmark of NCG

s	**Weight 3 genes**	**Weight 2 genes**	**HotNot2 Weight 2 genes**	**Weight 1 genes**	**HotNet2 Weight 1 genes**
0	53/73/72.6%	71/163/43.6%	55/118/46.6%	164/874/18.8%	98/490/20.0%
1	48/63/76.2%	61/133/45.9%	43/87/49.4%	152/714/21.3%	63/321/19.6%
2	40/51/78.4%	61/112/54.5%	46/80/57.5%	138/598/23.1%	51/243/21.0%
3	38/48/79.2%	52/92/56.5%	43/69/62.3%	135/520/26.0%	49/207/23.7%

*The first column corresponds to parameter *s*. For each situation, the A/B/C gives the sensitivity, the number of predicted genes and the accuracy of genes. The cancer gene benchmark is NCG cancer gene list.

Supplementary Table 11: Different weight genes of the 24 CovEx solutions with comparison benchmark of CGC

s	**Weight 3 genes**	**Weight 2 genes**	**HotNot2 Weight 2 genes**	**Weight 1 genes**	**HotNet2 Weight 1 genes**
0	46/73/63.0%	48/163/29.4%	39/118/33.1%	91/874/10.4%	54/490/11.0%
1	40/63/63.5%	44/133/33.1%	31/87/35.6%	85/714/11.9%	33/321/10.3%
2	35/51/68.6%	38/112/33.9%	30/80/37.5%	82/598/13.7%	27/243/11.1%
3	33/48/68.8%	33/92/35.9%	27/69/39.1%	82/520/15.8%	28/207/13.5%

*The cancer gene benchmark is CGC cancer gene list. The Meanings of values in the table are similar to those in Supplementary Table 10.

Supplementary Table 12: Different weight genes of the 24 CovEx solutions with comparison benchmark of 20/20 rule

s	**Weight 3 genes**	**Weight 2 genes**	**HotNot2 Weight 2 genes**	**Weight 1 genes**	**HotNet2 Weight 1 genes**
0	39/73/53.4%	28/163/17.2%	21/118/17.8%	22/874/2.5%	10/490/2.0%
1	33/63/52.4%	30/133/22.6%	21/87/24.1%	22/714/3.1%	8/321/2.5%
2	29/51/56.9%	26/112/23.2%	21/80/26.3%	28/598/4.7%	7/243/2.9%
3	27/48/56.3%	25/92/27.2%	20/69/29.0%	30/520/5.8%	9/207/4.3%

*The cancer gene benchmark is 20/20 rule cancer gene list. The Meanings of values in the table are similar to those in Supplementary Table 10.

Supplementary Table 13: CovEx networks for single cancer types obtained by consensus method A.
See Supplementary_Table_13

Supplementary Table 14: Comparison results of CovEx and HotNet2 for single cancer types with comparison benchmark of CGC

	NC	SC	AC	NH	SH	AH
BLCA	30	14	46.6%	147	33	22.4%
BRCA	116	46	39.7%	50	15	30.0%
COADREAD	19	12	63.2%	76	14	18.4%
GBM	43	19	44.2%	25	10	40.0%
HNSC	66	24	36.4%	93	15	16.1%
KIRC	76	28	36.8%	23	5	21.7%
LAML	73	31	42.5%	42	22	52.3%
LUAD	52	24	46.2%	240	29	12.1%
LUSC	27	16	59.3%	103	16	15.5%
OV	28	13	46.4%	25	8	32.0%
UCEC	20	16	80.0%	73	22	30.1%

*The first column corresponds to the cancer types. For each cancer type, the columns NC, SC, AC and NH, SH, AH correspond to the number of output genes, sensitivities and accuracies of CovEx and HotNet2, respectively. The cancer gene benchmark is CGC cancer gene list.

Supplementary Table 15: Comparison results of CovEx and HotNet2 for single cancer types with comparison benchmark of 20/20 rule

	NC	SC	AC	NH	SH	AH
BLCA	30	12	40.0%	147	17	11.6%
BRCA	116	32	27.6%	50	10	20.0%
COADREAD	19	11	57.9%	76	6	7.9%
GBM	43	15	34.9%	25	8	32.0%
HNSC	66	18	27.3%	93	8	8.6%
KIRC	76	19	25.0%	23	5	21.7%
LAML	73	23	31.5%	42	14	33.3%
LUAD	52	19	36.5%	240	13	5.4%
LUSC	27	12	44.4%	103	10	9.7%
OV	28	9	32.1%	25	4	16.0%
UCEC	20	13	65.0%	73	11	15.1%

*Each column has the same meaning as in Supplementary Table 14. The cancer gene benchmark is 20/20 rule cancer gene list.

Supplementary Table 16: Genes identified by CovEx for at least two cancer types. See Supplementary_Table_16

Supplementary Table 17: Proportion of NCG cancer genes of different weights of consensus method B

s	≥ 10	= 9	= 8	= 7	= 6	= 5
0	36/53/67.9%	11/20/55.0%	14/28/50.0%	3/10/30.0%	26/61/42.6%	15/42/35.7%
1	29/42/69.0%	8/12/66.7%	10/11/90.9%	3/7/42.9%	20/33/60.6%	20/41/48.8%
2	22/32/68.8%	6/9/66.7%	11/13/84.6%	3/7/42.9%	21/28/75.0%	18/34/52.9%
3	19/27/70.4%	9/13/69.2%	8/10/80.0%	4/6/66.7%	14/19/73.7%	22/29/75.9%

*The numbers in the first column corresponds to the parameter *s*. For each situation, the A/B/C gives the sensitivity, the number of predicted genes and the accuracy.

Supplementary Table 18: Accumulate proportion of NCG cancer genes of different weights of consensus method B

s	≥ 10	≥ 9	≥ 8	≥ 7	≥ 6	≥ 5
0	36/53/67.9%	47/73/64.4%	61/101/60.4%	64/111/57.7%	90/172/52.3%	105/214/49.1%
1	29/42/69.0%	37/54/68.5%	47/65/72.3%	50/72/69.4%	70/105/66.7%	90/146/61.6%
2	22/32/68.8%	28/41/68.3%	39/54/72.2%	42/61/68.9%	63/89/70.8%	81/123/65.9%
3	19/27/70.4%	28/40/70.0%	36/50/72.0%	40/56/71.4%	54/75/72.0%	76/104/73.1%

*The numbers in the first column corresponds to the parameter *s*. For each situation, the A/B/C gives the sensitivity, the number of predicted genes and the accuracy.

Supplementary Table 19: Genes with weights at least 5 identified by consensus method B

Weight	Genes
≥ 10	ADNP[2] AKT1(+)[4] APC(+)[4] ARID1A(+)[4] ATAD5[2] ATM(+)[4] BCL2L1[4] CCND1(+)[4] CCNE1(+)[4] CDK4(+)[4] CDKN2A(+)[4] CREBBP(+)[4] CRIPAK[4] CTNNB1(+)[4] DNMT3A(+)[4] EGFR(+)[4] ERBB2(+)[4] FBXW7(+)[4] INTS4[4] ITGB2 KAT6A(+)[4] KAT6B(+)[4] KDM6A(+)[4] KIT(+)[4] LILRB1(+)[4] LILRB2[4] MAP1A[4] MCL1[4] MDM2(+)[2] MDM4(+) MED12(+)[4] MED13[4] MLL2(+)[4] MLL3(+)[4] MTOR(+)[4] PARP10[4] PBRM1(+)[4] PIK3CA(+)[4] PIK3R1(+)[4] POLR2A[4] POM121C[2] PTPN11(+)[4] RB1(+)[4] RBMX(+)[4] SETD2(+)[4] SMC3(+)[2] SMG1[4] SRRM2[4] TLN1 TP53(+)[4] UPF2[4] VHL(+)[4] ZNF687(+)[4]
9	ANKRD17[4] BRD4(+)[4] EP300(+)[4] IKBKB ITGA2 KRAS(+)[4] MCM10 MLLT4(+)[4] MYC(+)[4] NAV1[4] NPM1(+)[4] NRAS(+)[4] RPGR(+)[4] RUNX1(+)[4] SMARCA4(+)[4] SUPT6H[4] TET2(+)[4] TNS1[3] WNK1[3] ZC3HC1
8	ABL1(+) ADAMTS1 ADRBK1 BAP1(+)[4] BUB3 CDKN1B(+) CFH CHD6(+)[4] CTCF(+)[4] DAG1 DDX11[4] DYSF EP400 FLT1(+) FLT3(+)[4] HPS4[2] IDH2(+)[4] IGF1R KDM5A(+)[2] PDGFRB(+) PTEN(+)[4] SHPRH[3] SMARCA2[3] TLR4(+)[4] USP2 USP36[3] WHSC1L1(+)[4] XPO1(+)[2]
7	ARHGEF1[2] BCL6(+)[2] CEP350 CHD4(+)[4] HCFC1 HIVEP1 NCOA3 NCOR1(+)[4] SPTAN1[4] SPTB[4]
6	ACACB ACTR3B AKAP13(+)[4] AP3D1 ARID2(+) BRCA1(+)[2] C3(+) CBX8[4] CD163 CDH1(+)[4] CEBPA(+)[4] CELSR3[2] COL5A1(+)[3] CR1 DDX50 DHX33 E2F3[4] EPPK1[4] EXOC7 EXT1(+) EZH2(+)[4] F8(+)[4] FBLN2 FLII G6PD[4] GATA3(+)[4] GIGYF2(+)[4] GOLGB1[2] GSTM3 HLA-DRB1[4] IDH1(+)[4] KIAA1549(+)[2] LAMA5 MAP3K1(+)[3] MAPK14 MECOM(+)[4] MYH11(+)[4] MYO1D MYO5B[2] NBPF1[3] NID2 NOTCH1(+)[4] NOTCH3(+)[4] OVGP1 PABPC1(+)[4] PIK3CG(+) PPP2R1A(+)[4] PROS1[4] PRRC2C[3] PTCH1(+) RIN2 SLC27A5 SMC1A(+)[4] SREBF2[2] ST5 STK11(+) TBL2 TRIP12[2] WT1(+)[4] ZBED4 ZMAT1
5	A2M AKAP3[3] ANKHD1 BCOR(+)[2] BRAF(+)[2] CBX1[2] CEP135[2] CIT[3] COL14A1[3] DNMT1(+)[3] DYRK4 ERBB4(+) FGFR3(+)[4] FOXA1(+)[4] IFT140 MAP1B[3] MAX(+)[4] MCC[3] MLL[4] MYCL1[4] NEDD9[3] NF1(+)[4] NFKB1 NOL12 NRXN2(+)[4] ORC1[2] PCNX PDGFRA(+)[3] PDXDC1(+) PLCE1[4] PLXNA1 RANBP2 RPS27L RRP12[3] SERPING1 SF3B1(+) SPTBN1[4] TCF3(+)[3] UBR5(+)[4] XRN1[2] ZNF217 ZNF844(+)

*Genes with '+' are NCG cancer genes. The number *s* in the bracket means that the gene was identified by consensus method B with (*s*-1)-modules filtered. In the situation of *s*-modules filtered case, genes with weight at least 5 were identified by consensus method B.

Supplementary Table 20: Networks constructed by consensus method B

Network index	Networks
1	A2M ABL1 ACACB ACTR3B ADAMTS1 ADNP ADRBK1 AKT1 ANKHD1 ANKRD17 AP3D1 APC ARHGEF1 ARID1A ARID2 ATAD5 ATM BAP1 BCL2L1 BCL6 BCOR BRAF BRCA1 BRD4 BUB3 C3 CBX1 CBX8 CCND1 CCNE1 CDK4 CDKN1B CDKN2A CEBPA CELSR3 CEP135 CFH CHD4 COL14A1 COL5A1 CR1 CREBBP CRIPAK CTCF CTNNB1 DAG1 DDX11 DDX50 DHX33 DNMT1 DNMT3A DYRK4 DYSF E2F3 EGFR EP300 EP400 EPPK1 ERBB2 ERBB4 EXOC7 EXT1 EZH2 F8 FBLN2 FBXW7 FGFR3 FLII FLT1 FLT3 FOXA1 G6PD GATA3 GIGYF2 GOLGB1 GSTM3 HCFC1 HLA-DRB1 HPS4 IDH1 IDH2 IFT140 IGF1R IKBKB ITGA2 ITGB2 KAT6A KAT6B KDM5A KDM6A KIAA1549 KIT KRAS LAMA5 LILRB1 LILRB2 MAP1A MAP1B MAP3K1 MAPK14 MCC MCL1 MCM10 MDM2 MDM4 MECOM MED12 MED13 MLL MLL2 MLL3 MLLT4 MTOR MYC MYH11 MYO1D MYO5B NAV1 NBPF1 NCOA3 NCOR1 NEDD9 NF1 NFKB1 NID2 NOL12 NOTCH1 NOTCH3 NPM1 NRAS NRXN2 ORC1 PABPC1 PARP10 PBRM1 PCNX PDGFRA PDGFRB PDXDC1 PIK3CA PIK3CG PIK3R1 PLCE1 PLXNA1 POLR2A POM121C PROS1 PRRC2C PTCH1 PTEN PTPN11 RB1 RIN2 RPGR RPS27L RRP12 RUNX1 SERPING1 SETD2 SF3B1 SHPRH SLC27A5 SMARCA2 SMARCA4 SMC1A SMC3 SMG1 SPTAN1 SPTB SPTBN1 SREBF2 ST5 STK11 SUPT6H TBL2 TCF3 TET2 TLN1 TP53 TRIP12 UBR5 UPF2 USP2 USP36 VHL WHSC1L1 WT1 XPO1 XRN1 ZBED4 ZC3HC1 ZMAT1 ZNF217 ZNF844
2	AKAP13 AKAP3 CDH1 CIT RANBP2
3	CEP350 HIVEP1
4	CD163 OVGP1 TNS1 WNK1
5	CHD6 TLR4
6	INTS4 PPP2R1A ZNF687
7	RBMX SRRM2
8	MAX MYCL1

Supplementary Table 21: CovEx results of both consensus methods with comparison benchmark of NCG

s	Weight 3 genes	Method A	Method B	Combined genes	Common genes
0	53/73/72.6%	124/236/52.5%	105/214/49.1%	135/288/46.9%	94/162/58.0%
1	48/63/76.2%	109/196/55.6%	90/146/61.6%	118/221/53.4%	83/124/66.9%
2	40/51/78.4%	101/163/62.0%	81/123/65.9%	109/184/59.2%	74/103/71.8%
3	38/48/79.2%	90/140/64.3%	76/104/73.1%	97/156/62.2%	71/90/78.9%
4	38/48/79.2%	82/126/65.1%	67/90/74.4%	87/137/63.5%	64/81/79.0%
5	35/42/83.3%	80/116/69.0%	62/81/76.5%	86/128/67.2%	58/72/80.6%
6	33/36/91.7%	71/98/72.4%	54/69/78.3%	78/110/70.9%	51/61/83.6%
7	32/35/91.4%	66/88/75.0%	51/61/83.6%	73/98/74.5%	48/55/87.3%

*The numbers in the first column corresponds to the parameter *s*. "Weight 3 genes" corresponds to weight 3 genes of consensus method A. "Method A", "Method B" correspond to consensus method A and B, respectively. "Combined genes", "Common genes" correspond to combined genes and common genes of results of consensus method A and B, respectively. For each situation, the A/B/C gives the sensitivity, the number of predicted genes and the accuracy of CovEx. The cancer gene benchmark is NCG cancer gene list.

Supplementary Table 22: CovEx results of both consensus methods with comparison benchmark of CGC

s	Weight 3 genes	Method A	Method B	Combined genes	Common genes
0	46/73/63.0%	94/236/39.8%	82/214/38.3%	103/288/35.8%	73/162/45.1%
1	40/63/63.5%	84/196/42.9%	71/146/48.6%	93/221/42.1%	63/124/50.8%
2	35/51/68.6%	73/163/44.8%	63/123/51.2%	81/184/44.0%	56/103/54.4%
3	33/48/68.8%	66/1401/47.1%	60/104/57.7%	73/156/46.8%	55/90/61.1%
4	33/48/68.8%	60/126/47.6%	52/90/57.8%	65/137/47.4%	49/81/60.5%
5	33/42/78.6%	60/116/51.7%	52/81/64.2%	65/128/50.8%	49/72/68.1%
6	33/36/91.7%	57/98/58.2%	47/69/68.1%	63/110/57.3%	45/61/73.8%
7	32/35/91.4%	56/88/63.6%	47/61/77.0%	62/98/63.3%	45/55/81.8%

*The cancer gene benchmark is CGC cancer gene list. The meanings of values in the table are similar to those in Supplementary Table 21.

Supplementary Table 23: CovEx results of both consensus methods with comparison benchmark of 20/20 rule

	Weight 3 genes	Method A	Method B	Combined genes	Common genes
0	39/73/53.4%	67/236/28.4%	63/214/29.4%	71/288/24.7%	59/162/36.4%
1	33/63/52.4%	63/196/32.1%	56/146/38.4%	67/221/30.3%	53/124/42.7%
2	29/51/56.9%	55/163/33.7%	52/123/42.3%	58/184/31.5%	50/103/48.5%
3	27/48/56.3%	52/140/37.1%	49/104/47.1%	56/156/35.9%	47/90/52.2%
4	27/48/56.3%	50/126/39.7%	42/90/46.7%	53/137/38.7%	41/81/50.6%
5	27/42/64.3%	50/116/43.1%	42/81/51.9%	53/128/41.4%	41/72/56.9%
6	27/36/75.0%	48/98/49.0%	39/69/56.5%	52/110/47.3%	38/61/62.3%
7	26/35/74.3%	47/88/53.4%	39/61/63.9%	51/98/52.0%	38/55/69.1%

*The cancer gene benchmark is 20/20 rule cancer gene list. The meanings of values in the table are similar to those in Supplementary Table 21.

Supplementary Table 24: Consensus results for 24 CovEx solutions of different runs

	Weight 3 genes	Method A	Method B
λ = 0	40/56/71.4%	107/199/53.8%	102/218/46.8%
	40/57/70.2%	106/198/53.5%	101/219/46.1%
	43/59/72.9%	108/199/54.3%	101/218/46.3%
λ = 1	38/52/73.1%	103/182/56.6%	97/195/49.7%
	38/52/73.1%	101/181/55.8%	99/198/50.0%
	38/52/73.1%	103/184/56.0%	97/198/49.0%
λ = 0, λ = 1	53/73/72.6%	124/236/52.5%	105/214/49.1%
	54/75/72.0%	121/232/52.2%	106/218/48.6%
	55/75/73.3%	126/237/53.2%	105/216/48.6%

*The dataset is pan-cancer dataset A. "Weight 3 genes" corresponds to weight 3 genes of consensus method A. "Method A", "Method B" correspond to consensus method A and B, respectively. "λ = 0", "λ = 1" correspond to consensus results for the 12 corresponding CovEx solutions. "λ = 0, λ = 1" corresponds to consensus results for all the 24 solutions. The minimum weights of consensus method B for all the 24 solutions and the 12 solutions (λ = 0 or λ = 1) were set as 5 and 3, respectively. For each situation, the A/B/C gives the sensitivity, the number of predicted genes and the accuracy of CovEx. The cancer gene benchmark is NCG cancer gene list.
