# Supplementary material for: Identification of driver modules in pan-cancer via coordinating coverage and exclusivity
Source: Oncotarget. 2017 Mar 21;8(22):36115–26. doi: 10.18632/oncotarget.16433 (PMC5482642; doi:10.18632/oncotarget.16433)
Supplement: Supplementary file 2 [file oncotarget-08-36115-s002.docx]

**Supplementary Table 9. The top enriched functional pathways or GO terms for some type 1 sub-networks constructed by consensus method A.**

| Type 1 network index : 1 | | | |
| --- | --- | --- | --- |
| number of nodes: | | 121 | |
| number of edges: | | 955 | |
| average node degree: | | 15.8 | |
| clustering coefficient: | | 0.603 | |
| expected number of edges: | | 347 | |
| PPI enrichment p-value: | | 0 | |
| Biological Process (GO) | | | |
| pathway ID | pathway description | count in gene set | false discovery rate |
| GO:0051338 | regulation of transferase activity | 40 | 5.64E-22 |
| GO:0043549 | regulation of kinase activity | 37 | 7.55E-22 |
| GO:0031399 | regulation of protein modification process | 48 | 1.25E-20 |
| GO:0051726 | regulation of cell cycle | 37 | 2.31E-18 |
| GO:0048468 | cell development | 45 | 8.45E-18 |
| Molecular Function (GO) | | | |
| pathway ID | pathway description | count in gene set | false discovery rate |
| GO:0005515 | protein binding | 74 | 2.74E-18 |
| GO:0044877 | macromolecular complex binding | 34 | 1.28E-13 |
| GO:0003682 | chromatin binding | 24 | 4.03E-13 |
| GO:0019899 | enzyme binding | 32 | 1.05E-09 |
| GO:0008134 | transcription factor binding | 19 | 1.15E-09 |
| Cellular Component (GO) | | | |
| pathway ID | pathway description | count in gene set | false discovery rate |
| GO:0005654 | nucleoplasm | 65 | 2.24E-23 |
| GO:0044428 | nuclear part | 70 | 1.41E-21 |
| GO:0031981 | nuclear lumen | 66 | 1.36E-20 |
| GO:0005634 | nucleus | 87 | 3.60E-18 |
| GO:0043233 | organelle lumen | 68 | 1.32E-17 |
| KEGG Pathways | | | |
| pathway ID | pathway description | count in gene set | false discovery rate |
| 5200 | Pathways in cancer | 31 | 1.32E-26 |
| 5206 | MicroRNAs in cancer | 22 | 4.40E-23 |
| 5214 | Glioma | 17 | 1.71E-22 |
| 5215 | Prostate cancer | 18 | 1.31E-21 |
| 5218 | Melanoma | 16 | 5.66E-20 |
| INTERPRO Protein Domains and Features | | | |
| pathway ID | pathway description | count in gene set | false discovery rate |
| IPR011009 | Protein kinase-like domain | 10 | 0.000688 |
| IPR016024 | Armadillo-type fold | 8 | 0.000688 |
| IPR011989 | Armadillo-like helical | 6 | 0.00915 |
| IPR018936 | Phosphatidylinositol 3/4-kinase, conserved site | 3 | 0.0177 |
| IPR000403 | Phosphatidylinositol 3-/4-kinase, catalytic domain | 3 | 0.0291 |
| IPR017441 | Protein kinase, ATP binding site | 7 | 0.0291 |
|  | | | |
| Type 1 network index : 5 | | | |
| number of nodes: | | 11 | |
| number of edges: | | 18 | |
| average node degree: | | 3.27 | |
| clustering coefficient: | | 0.826 | |
| expected number of edges: | | 4 | |
| PPI enrichment p-value: | | 2.38e-07 | |
| Biological Process (GO) | | | |
| pathway ID | pathway description | count in gene set | false discovery rate |
| GO:0048732 | gland development | 6 | 0.000251 |
| GO:0007220 | Notch receptor processing | 3 | 0.000905 |
| GO:0001655 | urogenital system development | 5 | 0.00092 |
| GO:0030900 | forebrain development | 5 | 0.00149 |
| GO:0006366 | transcription from RNA polymerase II promoter | 6 | 0.00187 |
| Molecular Function (GO) | | | |
| pathway ID | pathway description | count in gene set | false discovery rate |
| GO:0046982 | protein heterodimerization activity | 4 | 0.0155 |
| Cellular Component (GO) | | | |
| pathway ID | pathway description | count in gene set | false discovery rate |
| GO:0031981 | nuclear lumen | 9 | 0.00329 |
| GO:0005654 | nucleoplasm | 8 | 0.0033 |
| GO:0005634 | nucleus | 10 | 0.013 |
| GO:0009898 | cytoplasmic side of plasma membrane | 3 | 0.013 |
| GO:0044446 | intracellular organelle part | 10 | 0.0255 |
| KEGG Pathways | | | |
| pathway ID | pathway description | count in gene set | false discovery rate |
| 05206 | MicroRNAs in cancer | 6 | 1.35e-08 |
| 04320 | Dorso-ventral axis formation | 3 | 2.54e-05 |
| 04919 | Thyroid hormone signaling pathway | 4 | 2.9e-05 |
| 04330 | Notch signaling pathway | 3 | 0.000133 |
| 04010 | MAPK signaling pathway | 4 | 0.000326 |
|  | | | |
| Type 1 network index : 8 | | | |
| number of nodes: | | 8 | |
| number of edges: | | 10 | |
| average node degree: | | 2.5 | |
| clustering coefficient: | | 0.883 | |
| expected number of edges: | | 1 | |
| PPI enrichment p-value: | | 7.82e-07 | |
| Biological Process (GO) | | | |
| pathway ID | pathway description | count in gene set | false discovery rate |
| GO:0000956 | nuclear-transcribed mRNA catabolic process | 4 | 0.00285 |
| GO:0000184 | nuclear-transcribed mRNA catabolic process, nonsense-mediated decay | 3 | 0.0149 |
| Molecular Function (GO) | | | |
| pathway ID | pathway description | count in gene set | false discovery rate |
| GO:0003723 | RNA binding | 6 | 0.0138 |
| GO:0044822 | poly(A) RNA binding | 5 | 0.0485 |
| Cellular Component (GO) | | | |
| pathway ID | pathway description | count in gene set | false discovery rate |
| GO:0005829 | cytosol | 7 | 0.0164 |
| KEGG Pathways | | | |
| pathway ID | pathway description | count in gene set | false discovery rate |
| 03013 | RNA transport | 3 | 0.00617 |
| 03015 | mRNA surveillance pathway | 2 | 0.046 |
| 03018 | RNA degradation | 2 | 0.046 |
|  | | | |
| Type 1 network index : 13 | | | |
| number of nodes: | | 8 | |
| number of edges: | | 10 | |
| average node degree: | | 2.5 | |
| clustering coefficient: | | 0.713 | |
| expected number of edges: | | 2 | |
| PPI enrichment p-value: | | 8.14e-06 | |
| Biological Process (GO) | | | |
| pathway ID | pathway description | count in gene set | false discovery rate |
| GO:0007155 | cell adhesion | 6 | 0.000909 |
| GO:0030030 | cell projection organization | 6 | 0.000909 |
| GO:0000904 | cell morphogenesis involved in differentiation | 5 | 0.00351 |
| GO:0031175 | neuron projection development | 5 | 0.00351 |
| GO:0042060 | wound healing | 5 | 0.00351 |
| Molecular Function (GO) | | | |
| pathway ID | pathway description | count in gene set | false discovery rate |
| GO:0001948 | glycoprotein binding | 5 | 5.84e-08 |
| GO:0050839 | cell adhesion molecule binding | 4 | 0.000207 |
| GO:0032403 | protein complex binding | 5 | 0.000715 |
| GO:0005515 | protein binding | 8 | 0.00374 |
| GO:0043394 | proteoglycan binding | 2 | 0.011 |
| Cellular Component (GO) | | | |
| pathway ID | pathway description | count in gene set | false discovery rate |
| GO:0005925 | focal adhesion | 4 | 0.00352 |
| GO:0008305 | integrin complex | 2 | 0.0109 |
| GO:0070062 | extracellular exosome | 6 | 0.0153 |
| GO:0043235 | receptor complex | 3 | 0.0175 |
| GO:0005901 | caveola | 2 | 0.0378 |
| KEGG Pathways | | | |
| pathway ID | pathway description | count in gene set | false discovery rate |
| 04510 | Focal adhesion | 5 | 1.4e-06 |
| 04015 | Rap1 signaling pathway | 4 | 9.35e-05 |
| 04611 | Platelet activation | 3 | 0.0012 |
| 05205 | Proteoglycans in cancer | 3 | 0.00461 |
| 04151 | PI3K-Akt signaling pathway | 3 | 0.00948 |
