# Supplementary material for: Identification of driver modules in pan-cancer via coordinating coverage and exclusivity
Source: Oncotarget. 2017 Mar 21;8(22):36115–26. doi: 10.18632/oncotarget.16433 (PMC5482642; doi:10.18632/oncotarget.16433)
Supplement: Supplementary file 3 [file oncotarget-08-36115-s003.docx]

**Supplementary Table 13. CovEx networks for single cancer types obtained by consensus method A.**

| Cancer type | Network index | Core networks  (Weight-3 genes) | Extended networks  (Weight-2 genes) | Linkers  (Weight-2 genes) |
| --- | --- | --- | --- | --- |
| BLCA | 1 | ARAP1 ARID1A ATM FGFR3 HNRNPA1 LUC7L2 MDM2 SMARCA2 TP53 | ACTL6A ADHFE1 CDKN1A CREBBP CUL1 E2F3 ELP2 EP300 KIAA1377 MYC NFRKB NSD1 ZFYVE9 |  |
|  | 2 | KDM6A MLL2 | ARHGAP32 |  |
|  | 3 | KDM5A RB1 | MLL3 |  |
|  | 4 |  | ACTL6A(1) EXOC1 MYC(1) TRIO |  |
|  | 5 |  | CREBBP(1) EP300(1) |  |
|  | 6 |  | CDKN1A(1) E2F3(1) |  |
| BRCA | 1 | AKT1 ARID1A ATM CCND1 CDH1 CTCF ERBB2 GATA3 IGF1R IKBKB KDM6A MAP3K1 MAPK14 MCC MDM4 MLL3 MYB MYC NCOA3 NCOR1 PIK3CA PJA2 POLR2A PTEN RBMX SMARCA4 TLN1 TP53 TSC22D1 UBE2L6 | ABCA1 ARHGEF1 ARID2 BLCAP CARM1 CBR1 CCDC50 CDKN2A CHD4 CIRBP CRTAP EGFR EPPK1 F8 FLNB HIPK2 HLA-DRB1 HSPA8 ILF2 INSR ITGA2 ITGB4 ITPR1 KRAS MAP2K4 MCL1 NEDD9 NOS3 NRAS OTUD4 PAXIP1 PLCE1 PLXNA1 PRKAR1A PTPN14 RPGR RPS27L SAAL1 SETD2 SF3B1 SH2B3 SMYD3 SPEN SPTB TARBP1 TBL1XR1 TBP TNS1 TRIM28 TRIP12 UFC1 USP36 WDR33 WHSC1L1 XPO1 | MDM2 RUNX1 |
|  | 2 | AXL PIK3R1 |  |  |
|  | 3 | BRCA1 CCNE1 RB1 | BACH1 E2F3 EXT1 | MDM2 |
|  | 4 | KAT6A KAT6B |  | RUNX1 |
|  | 5 | FOXA1 TLE1 | POLB | RUNX1 |
|  | 6 |  | CBX1 RIF1 SUV420H2 |  |
|  | 7 |  | INTS4 ZNF687 |  |
|  | 8 |  | SH3D19 SMYD3(1) WHSC1L1(1) |  |
|  | 9 |  | EPPK1(1) KRAS(1) MLLT4 NRAS(1) NRXN2 |  |
|  | 10 |  | CBFB POLB(5) RUNX1(1,4,5) ZNF217 |  |
|  | 11 |  | CHD4(1) USP36(1) |  |
|  | 12 |  | TNS1(1) WNK1 |  |
|  | 13 |  | BCL2L1 CASP8 MCL1(1) MDM2(1,3) MZF1 |  |
|  | 14 |  | TRIM28(1) XPO1(1) |  |
|  | 15 |  | EGFR(1) SEPP1 |  |
|  | 16 |  | MAP1A RPGR(1) |  |
|  | 17 |  | CRTAP(1) TARBP1(1) |  |
| COADREAD | 1 | APC EFTUD2 RABGAP1 SEC31A | AP1M1 CUX1 |  |
|  | 2 | ARID1A ATM CREBBP TP53 | RB1 SMAD2 |  |
|  | 3 | CTNNB1 TCF7L2 |  |  |
|  | 4 | KRAS NRAS PIK3CG | BRAF SPTBN1 |  |
| GBM | 1 | BRAF CDK4 CDKN2A CHEK2 EGFR EXOC7 ITGB2 MDM2 OGDH PIK3CA PIK3R1 PTEN PTPN11 RB1 TP53 | ABL1 APBB1 CDC27 CDC42EP1 CSRP2BP DDX56 ENC1 ITGAM LZTR1 MAPK8IP1 MCM3 MDM4 MYO1D NEDD4 NF1 PIK3CG RPL22 SYNJ2 TFDP1 TGFBRAP1 |  |
|  | 2 |  | CEP152 EPB41L3 |  |
|  | 3 |  | IDH1 PSAT1 |  |
|  | 4 |  | ANKRD17 NUP98 POM121C |  |
|  | 5 |  | BCOR LZTR1(1) MCM3(1) |  |
|  | 6 |  | NF1(1) TGFBRAP1(1) |  |
| HNSC | 1 | ARRB1 BCL2L1 CASP8 CLSPN CREBBP CYLD EP300 HRAS MTA2 NSD1 RAC1 TP53 | ATAD5 B2M BTK CCND1 FGFR1 GAK IKBKB LRSAM1 MYC NFATC1 PMS1 PSMA1 RB1 SMARCA2 SREBF2 STAT2 TICAM1 TRAF3 UPF2 ZNF175 | EGFR |
|  | 2 | PIK3CA PIK3R1 | ARHGEF1 PLCE1 | LFNG |
|  | 3 | NOTCH1 NOTCH3 | FBXW7 LTBP1 PTPRC TLN1 | LFNG |
|  | 4 | ASH2L MLL2 |  |  |
|  | 5 | PPFIA1 PPFIA4 | EIF4G1 | EGFR |
|  | 6 |  | CDC7 CDKN2A |  |
|  | 7 |  | ANKHD1 CDK6 KIAA2026 ZNF609 |  |
|  | 8 |  | AMBRA1 MAP1B |  |
|  | 9 |  | EPB41L3 SPTBN1 |  |
|  | 10 |  | CFH SERPING1 |  |
|  | 11 |  | AR ATN1 B2M(1) EGFR(1,5) HLA-A LILRB1 MYC(1) SMARCA2(1) |  |
|  | 12 |  | MAPK1 RB1(1) |  |
| KIRC | 1 | APC ATM BAP1 BARD1 BRD4 MLL2 MLL3 MTOR PABPC1 PBRM1 PDGFRA PIK3CA PLAUR PTEN SETD2 SMARCA4 SPEN TCEB1 TCF3 TP53 VHL | ADAP1 AGK AQR ARCN1 ARID1A BCL6 CLIP1 CPSF1 CUL2 CUL9 DNAJC14 DOCK7 EGFR KAT7 KDM5B KDM5C MDM4 MED13 MYH11 NCOR1 NFE2L2 NUP160 ORC1 PJA2 RANBP2 RHEB RIF1 RPL5 STAT1 TECPR1 TOM1L2 UBR5 VAPB ZC3H12A ZNF407 |  |
|  | 2 |  | MTMR4 RGS3 |  |
|  | 3 |  | CCDC88B CNTROB |  |
|  | 4 |  | GOLGA6L5 PCSK5 YLPM1 |  |
|  | 5 |  | DDX39B RBM39 |  |
|  | 6 |  | ERBB4 MARCH2 |  |
|  | 7 |  | CCDC120 CENPE |  |
|  | 8 |  | MED13(1) POLR2B |  |
|  | 9 |  | CUL3 NFE2L2(1) |  |
|  | 10 |  | IFT140 RANBP2(1) |  |
|  | 11 |  | DIP2C ZNF407(1) |  |
|  | 12 |  | DENND4A STAT1(1) |  |
|  | 13 |  | AVIL EGFR(1) MYO18A |  |
| LAML | 1 | ABL1 ABTB1 ADRBK1 AKAP13 ASXL1 CALR CEBPA DAG1 DDX41 DNMT3A EED ELL EZH2 FLT3 FRYL HIVEP1 KDM6A KIT KRAS LNX1 MCM10 MLLT4 NPM1 NRAS PTPN11 RUNX1 SELENBP1 SMARCA2 SMC1A SMC3 SPEN SREBF2 STAG2 TP53 U2AF1 WT1 | ARHGEF1 ARHGEF6 ARID2 ATXN7L1 BICD1 CBX7 CCDC136 CD74 COPS2 CUL3 DIS3 E2F8 GIGYF2 HNRNPK IDH1 IDH2 IL1R1 MAX MYC NF1 PARD3 PRPF8 RAD21 RIN1 SHC1 SHQ1 SMG1 TET2 THRAP3 TOP3B TRIO VCAM1 ZBED4 |  |
|  | 2 |  | GIGYF2(1) NAV1 POM121C TBC1D4 TET2(1) |  |
|  | 3 |  | FAM65A SHC1(1) |  |
|  | 4 |  | IDH1(1) IDH2(1) |  |
| LUAD | 1 | ADNP ARID1A ARID2 ATM CTNNB1 DNMT1 E2F1 EGFR ERBB2 HDAC4 KEAP1 KRAS MDM2 RB1 SMARCA4 TP53 ZNF23 | ARNT ARRB1 BRAF CDK4 CDKN2A CLASP2 CRIPAK DDX11 DVL3 EIF4G3 FHOD1 FLT1 MDM4 NIPBL NUP214 SETD7 SMARCA2 TTC37 WRN ZNF407 ZNF92 | PTPRB |
|  | 2 | HCFC1 MLL3 | CHD8 MLL2 NCOA6 | PTPRB |
|  | 3 |  | EXPH5 MCL1 MYO5A |  |
|  | 4 |  | CR1 ITGAX |  |
|  | 5 |  | NF1 SMAD4 |  |
|  | 6 |  | CDK4(1) CDKN2A(1) |  |
|  | 7 |  | CHD8(2) MLL2(2) |  |
|  | 8 |  | CRIPAK(1) ZNF83 |  |
|  | 9 |  | ZNF407(1) ZNF92(1) |  |
| LUSC | 1 | KEAP1 NFE2L2 | MDM2 |  |
|  | 2 | KDM5A RB1 | CCND1 KLHDC2 PIK3CA |  |
|  | 3 | CDKN2A MLL3 NCOA6 | CDK6 MLL2 |  |
|  | 4 |  | BAP1 PPP2R1A WHSC1L1 |  |
|  | 5 |  | CCND1(2) FANCB G6PD MDM2(1) MLL2(3) NCOA1 NF1 PIK3CG PLK3 SP1 TP53 TSSK6 UBC WNK1 |  |
| OV | 1 | ERCC2 KBTBD7 TP53 WRN | DNMBP HDAC6 IFRD2 LRIF1 SPG7 SSX2IP STK4 WDR33 WIPF1 | UBA1 |
|  | 2 | AKT1 CCND1 CCNE1 CDKN1B FBXW7 | CHD4 MYC | UBA1 |
|  | 3 | BRAF KRAS | KDM2A RAP1GAP STK11 |  |
|  | 4 |  | DHX33 MLL3 |  |
|  | 5 |  | LRIF1(1) SPG7(1) |  |
|  | 6 |  | CHD4(2) MYC(2) |  |
|  | 7 |  | DNMBP(1) WIPF1(1) |  |
| UCEC | 1 | AKT1 CELSR3 PIK3CA PIK3R1 PTEN PTPRB TP53 | ABL1 ARHGAP26 ARHGEF19 BRAF EEF1D RANBP2 | ERBB2 PPP2R1A |
|  | 2 | CTNNB1 FBXW7 KRAS | DVL3 MYC | ERBB2 PPP2R1A |

*The networks containing no weight 3 genes are type 2 networks. The genes with parentheses belong to the type 1 networks and the number in the parentheses corresponds to the type 1 network index.
