# Supplementary material for: Identification of driver modules in pan-cancer via coordinating coverage and exclusivity
Source: Oncotarget. 2017 Mar 21;8(22):36115–26. doi: 10.18632/oncotarget.16433 (PMC5482642; doi:10.18632/oncotarget.16433)
Supplement: Supplementary file 4 [file oncotarget-08-36115-s004.docx]

**Supplementary Table 16. Genes identified by CovEx for at least two cancer types.**

|  | C1 | C2 | C3 | C4 | C5 | C6 | C7 | C8 | C9 | C10 | C11 | C12 | C13 | C14 |
| --- | --- | --- | --- | --- | --- | --- | --- | --- | --- | --- | --- | --- | --- | --- |
| TP53 | 3 | 111 | 11 | 3 | 3 | 3 | 3 | 3 | 3 | 3 | 3 | 2 | 3 | 3 |
| RB1 | 3 | 111 | 7 | 3 | 3 | 2 | 3 | 2 | 0 | 0 | 3 | 3 | 0 | 0 |
| KRAS | 3 | 111 | 6 | 0 | 2 | 3 | 0 | 0 | 0 | 3 | 3 | 0 | 3 | 3 |
| MLL3 | 3 | 111 | 6 | 2 | 3 | 0 | 0 | 0 | 3 | 0 | 3 | 3 | 2 | 0 |
| MYC | 3 | 111 | 6 | 2 | 3 | 0 | 0 | 2 | 0 | 2 | 0 | 0 | 2 | 2 |
| PIK3CA | 3 | 111 | 6 | 0 | 3 | 0 | 3 | 3 | 3 | 0 | 0 | 2 | 0 | 3 |
| ARID1A | 3 | 111 | 5 | 3 | 3 | 3 | 0 | 0 | 2 | 0 | 3 | 0 | 0 | 0 |
| ATM | 3 | 111 | 5 | 3 | 3 | 3 | 0 | 0 | 3 | 0 | 3 | 0 | 0 | 0 |
| BRAF | 2 | 111 | 5 | 0 | 0 | 2 | 3 | 0 | 0 | 0 | 2 | 0 | 3 | 2 |
| CDKN2A | 3 | 111 | 5 | 0 | 2 | 0 | 3 | 2 | 0 | 0 | 2 | 3 | 0 | 0 |
| EGFR | 3 | 111 | 5 | 0 | 2 | 0 | 3 | 2 | 2 | 0 | 3 | 0 | 0 | 0 |
| MDM2 | 3 | 111 | 5 | 3 | 2 | 0 | 3 | 0 | 0 | 0 | 3 | 2 | 0 | 0 |
| MLL2 | 3 | 111 | 5 | 3 | 0 | 0 | 0 | 3 | 3 | 0 | 2 | 2 | 0 | 0 |
| CCND1 | 3 | 111 | 4 | 0 | 3 | 0 | 0 | 2 | 0 | 0 | 0 | 2 | 3 | 0 |
| MDM4 | 3 | 111 | 4 | 0 | 3 | 0 | 2 | 0 | 2 | 0 | 2 | 0 | 0 | 0 |
| NF1 | 2 | 111 | 4 | 0 | 0 | 0 | 2 | 0 | 0 | 2 | 2 | 2 | 0 | 0 |
| PIK3R1 | 3 | 111 | 4 | 0 | 3 | 0 | 3 | 3 | 0 | 0 | 0 | 0 | 0 | 3 |
| PTEN | 2 | 111 | 4 | 0 | 3 | 0 | 3 | 0 | 3 | 0 | 0 | 0 | 0 | 3 |
| SMARCA2 | 3 | 000 | 4 | 3 | 0 | 0 | 0 | 2 | 0 | 3 | 2 | 0 | 0 | 0 |
| ABL1 | 3 | 111 | 3 | 0 | 0 | 0 | 2 | 0 | 0 | 3 | 0 | 0 | 0 | 2 |
| AKT1 | 3 | 111 | 3 | 0 | 3 | 0 | 0 | 0 | 0 | 0 | 0 | 0 | 3 | 3 |
| ARHGEF1 | 2 | 000 | 3 | 0 | 2 | 0 | 0 | 2 | 0 | 2 | 0 | 0 | 0 | 0 |
| ARID2 | 3 | 111 | 3 | 0 | 2 | 0 | 0 | 0 | 0 | 2 | 3 | 0 | 0 | 0 |
| CREBBP | 3 | 111 | 3 | 2 | 0 | 3 | 0 | 3 | 0 | 0 | 0 | 0 | 0 | 0 |
| CTNNB1 | 3 | 111 | 3 | 0 | 0 | 3 | 0 | 0 | 0 | 0 | 3 | 0 | 0 | 3 |
| ERBB2 | 3 | 111 | 3 | 0 | 3 | 0 | 0 | 0 | 0 | 0 | 3 | 0 | 0 | 2 |
| FBXW7 | 3 | 111 | 3 | 0 | 0 | 0 | 0 | 2 | 0 | 0 | 0 | 0 | 3 | 3 |
| KDM6A | 3 | 111 | 3 | 3 | 3 | 0 | 0 | 0 | 0 | 3 | 0 | 0 | 0 | 0 |
| NRAS | 3 | 111 | 3 | 0 | 2 | 3 | 0 | 0 | 0 | 3 | 0 | 0 | 0 | 0 |
| PIK3CG | 2 | 100 | 3 | 0 | 0 | 3 | 2 | 0 | 0 | 0 | 0 | 2 | 0 | 0 |
| SMARCA4 | 3 | 111 | 3 | 0 | 3 | 0 | 0 | 0 | 3 | 0 | 3 | 0 | 0 | 0 |
| SPEN | - | 010 | 3 | 0 | 2 | 0 | 0 | 0 | 3 | 3 | 0 | 0 | 0 | 0 |
| APC | 3 | 111 | 2 | 0 | 0 | 3 | 0 | 0 | 3 | 0 | 0 | 0 | 0 | 0 |
| ARRB1 | - | 000 | 2 | 0 | 0 | 0 | 0 | 3 | 0 | 0 | 2 | 0 | 0 | 0 |
| BAP1 | 2 | 111 | 2 | 0 | 0 | 0 | 0 | 0 | 3 | 0 | 0 | 2 | 0 | 0 |
| BCL2L1 | 3 | 000 | 2 | 0 | 2 | 0 | 0 | 3 | 0 | 0 | 0 | 0 | 0 | 0 |
| CASP8 | - | 111 | 2 | 0 | 2 | 0 | 0 | 3 | 0 | 0 | 0 | 0 | 0 | 0 |
| CCNE1 | 3 | 110 | 2 | 0 | 3 | 0 | 0 | 0 | 0 | 0 | 0 | 0 | 3 | 0 |
| CDK4 | 3 | 110 | 2 | 0 | 0 | 0 | 3 | 0 | 0 | 0 | 2 | 0 | 0 | 0 |
| CDK6 | 2 | 110 | 2 | 0 | 0 | 0 | 0 | 2 | 0 | 0 | 0 | 2 | 0 | 0 |
| CHD4 | 2 | 100 | 2 | 0 | 2 | 0 | 0 | 0 | 0 | 0 | 0 | 0 | 2 | 0 |
| CUL3 | - | 000 | 2 | 0 | 0 | 0 | 0 | 0 | 2 | 2 | 0 | 0 | 0 | 0 |
| DVL3 | - | 000 | 2 | 0 | 0 | 0 | 0 | 0 | 0 | 0 | 2 | 0 | 0 | 2 |
| E2F3 | - | 000 | 2 | 2 | 2 | 0 | 0 | 0 | 0 | 0 | 0 | 0 | 0 | 0 |
| EP300 | 3 | 111 | 2 | 2 | 0 | 0 | 0 | 3 | 0 | 0 | 0 | 0 | 0 | 0 |
| EPB41L3 | - | 100 | 2 | 0 | 0 | 0 | 2 | 2 | 0 | 0 | 0 | 0 | 0 | 0 |
| IDH1 | 2 | 111 | 2 | 0 | 0 | 0 | 2 | 0 | 0 | 2 | 0 | 0 | 0 | 0 |
| IKBKB | 3 | 010 | 2 | 0 | 3 | 0 | 0 | 2 | 0 | 0 | 0 | 0 | 0 | 0 |
| KDM5A | 2 | 110 | 2 | 3 | 0 | 0 | 0 | 0 | 0 | 0 | 0 | 3 | 0 | 0 |
| KEAP1 | - | 100 | 2 | 0 | 0 | 0 | 0 | 0 | 0 | 0 | 3 | 3 | 0 | 0 |
| MCL1 | 3 | 000 | 2 | 0 | 2 | 0 | 0 | 0 | 0 | 0 | 2 | 0 | 0 | 0 |
| MLLT4 | 3 | 110 | 2 | 0 | 2 | 0 | 0 | 0 | 0 | 3 | 0 | 0 | 0 | 0 |
| NCOA6 | - | 000 | 2 | 0 | 0 | 0 | 0 | 0 | 0 | 0 | 2 | 3 | 0 | 0 |
| NCOR1 | 2 | 111 | 2 | 0 | 3 | 0 | 0 | 0 | 2 | 0 | 0 | 0 | 0 | 0 |
| NFE2L2 | 2 | 111 | 2 | 0 | 0 | 0 | 0 | 0 | 2 | 0 | 0 | 3 | 0 | 0 |
| NSD1 | - | 110 | 2 | 2 | 0 | 0 | 0 | 3 | 0 | 0 | 0 | 0 | 0 | 0 |
| PJA2 | - | 000 | 2 | 0 | 3 | 0 | 0 | 0 | 2 | 0 | 0 | 0 | 0 | 0 |
| PLCE1 | 2 | 000 | 2 | 0 | 2 | 0 | 0 | 2 | 0 | 0 | 0 | 0 | 0 | 0 |
| POM121C | 3 | 000 | 2 | 0 | 0 | 0 | 2 | 0 | 0 | 2 | 0 | 0 | 0 | 0 |
| PPP2R1A | 2 | 111 | 2 | 0 | 0 | 0 | 0 | 0 | 0 | 0 | 0 | 2 | 0 | 2 |
| PTPN11 | 3 | 111 | 2 | 0 | 0 | 0 | 3 | 0 | 0 | 3 | 0 | 0 | 0 | 0 |
| PTPRB | - | 110 | 2 | 0 | 0 | 0 | 0 | 0 | 0 | 0 | 2 | 0 | 0 | 3 |
| RANBP2 | 2 | 010 | 2 | 0 | 0 | 0 | 0 | 0 | 2 | 0 | 0 | 0 | 0 | 2 |
| RIF1 | - | 000 | 2 | 0 | 2 | 0 | 0 | 0 | 2 | 0 | 0 | 0 | 0 | 0 |
| RUNX1 | 2 | 111 | 2 | 0 | 2 | 0 | 0 | 0 | 0 | 3 | 0 | 0 | 0 | 0 |
| SETD2 | 2 | 111 | 2 | 0 | 2 | 0 | 0 | 0 | 3 | 0 | 0 | 0 | 0 | 0 |
| SPTBN1 | - | 000 | 2 | 0 | 0 | 2 | 0 | 2 | 0 | 0 | 0 | 0 | 0 | 0 |
| SREBF2 | 3 | 000 | 2 | 0 | 0 | 0 | 0 | 2 | 0 | 3 | 0 | 0 | 0 | 0 |
| TLN1 | 3 | 000 | 2 | 0 | 3 | 0 | 0 | 2 | 0 | 0 | 0 | 0 | 0 | 0 |
| TRIO | - | 000 | 2 | 2 | 0 | 0 | 0 | 0 | 0 | 2 | 0 | 0 | 0 | 0 |
| WDR33 | - | 000 | 2 | 0 | 2 | 0 | 0 | 0 | 0 | 0 | 0 | 0 | 2 | 0 |
| WHSC1L1 | 3 | 110 | 2 | 0 | 2 | 0 | 0 | 0 | 0 | 0 | 0 | 2 | 0 | 0 |
| WNK1 | 2 | 000 | 2 | 0 | 2 | 0 | 0 | 0 | 0 | 0 | 0 | 2 | 0 | 0 |
| WRN | - | 110 | 2 | 0 | 0 | 0 | 0 | 0 | 0 | 0 | 2 | 0 | 3 | 0 |
| ZNF407 | - | 000 | 2 | 0 | 0 | 0 | 0 | 0 | 2 | 0 | 2 | 0 | 0 | 0 |

*The ‘3’, ‘2’ in column C1 means that the gene is a weight 3, weight 2 gene identified by CovEx for the pan-cancer dataset, respectively. The three numbers in column C2 indicate whether the gene belongs to the cancer gene list of NCG, CGC or 20/20 rule cancer genes, orderly. If the gene belongs to the corresponding cancer gene list, the number is 1, and 0 otherwise. The number in column C3 is the number of cancer types that the gene was identified by CovEx. The columns C4, C5, to C14 correspond to the cancer types BLCA, BRCA, COADREAD, GBM, HNSC, KIRC, LAML, LUAD, LUSC, OV and UCEC, orderly. The ‘3’ and ‘2’ in these columns mean that the gene was identified as a weight 3, weight 2 gene, respectively.
